# Systemic Homeostasis in Metabolome, Ionome, and Microbiome of Wild Yellowfin Goby in Estuarine Ecosystem

**DOI:** 10.1038/s41598-018-20120-x

**Published:** 2018-02-22

**Authors:** Feifei Wei, Kenji Sakata, Taiga Asakura, Yasuhiro Date, Jun Kikuchi

**Affiliations:** 1 RIKEN Center for Sustainable Resource Science, 1-7-22 Suehiro-cho, Tsurumi-ku, Yokohama 235-0045 Japan; 2 Graduate School of Medical Life Science, Yokohama City University, 1-7-29 Suehiro-cho, Tsurumi-ku, Yokohama 230-0045 Japan; 3 Graduate School of Bioagricultural Sciences and School of Agricultural Sciences, Nagoya University, 1 Furo-cho, Chikusa-ku, Nagoya 464-8601 Japan

## Abstract

Data-driven approaches were applied to investigate the temporal and spatial changes of 1,022 individuals of wild yellowfin goby and its potential interaction with the estuarine environment in Japan. Nuclear magnetic resonance (NMR)-based metabolomics revealed that growth stage is a primary factor affecting muscle metabolism. Then, the metabolic, elemental and microbial profiles of the pooled samples generated according to either the same habitat or sampling season as well as the river water and sediment samples from their habitats were measured using NMR spectra, inductively coupled plasma optical emission spectrometry and next-generation 16 S rRNA gene sequencing. Hidden interactions in the integrated datasets such as the potential role of intestinal bacteria in the control of spawning migration, essential amino acids and fatty acids synthesis in wild yellowfin goby were further extracted using correlation clustering and market basket analysis-generated networks. Importantly, our systematic analysis of both the seasonal and latitudinal variations in metabolome, ionome and microbiome of wild yellowfin goby pointed out that the environmental factors such as the temperature play important roles in regulating the body homeostasis of wild fish.

## Introduction

Great achievements in science and technology are correlated with the development of approaches able to solve major open problems in each academic field. The technological advances in high-throughput chemical fingerprinting analytic techniques such as nuclear magnetic resonance (NMR)-based metabolomics enable researchers to prospectively understand the complex interactions within ecological systems simultaneously from a holistic view^1–7^. It is believed that the rapidly accumulating large-scale molecular-biological data such as metagenomes and metabolomes provide enough information to comprehend complicated global biological and ecological systems in a data-driven manner. More importantly, developments in data preprocessing^8,9^ and signal integration approaches^10,11^ allow to extract useful information and establish simulation models to predict ecosystem dynamics on the basis of the high dimensionality and large data sets derived from diverse components. Therefore, big data analysis along with bioinformatics tools and computational technologies provides a powerful approach to elucidate the most challenging questions such as to establish an integrated model of the processes constituting and maintaining in a dynamic fashion the state of the cells/organisms as well as the ecosystem^12^.

The environmental impact on ocean wildlife represents an emerging concern^13–15^. The yellowfin goby (*Acanthogobius flavimanus*) is a common benthic inhabitant of estuarine mudflat native to northern Asia and Japan^16,17^. The yellowfin goby is strongly sensitive to environmental variables influenced by coastal conditions and upland freshwater drainage, and thus may serve as a promising probe to evaluate the aquatic ecosystem in a manner similar to earthworms^18^, marine mussels^19^, and fathead minnows^20^. Metabolites are the end products of cellular regulatory processes, and their levels can be considered as the ultimate response of biological systems to both endogenous and environmental influences^21–23^. In addition, there is growing evidence that gut microbiota plays a predominant role in regulating multiple host metabolic pathways^24,25^. Differences in surrounding environmental conditions are likely to impact the health and characteristics of wildlife through the host-microbial metabolic interaction. Systematic analysis of the chemical dynamics of environment, host, and microbiome interaction will shed light on understanding the environmental homeostasis within an ecosystem.

**Figure 1:**
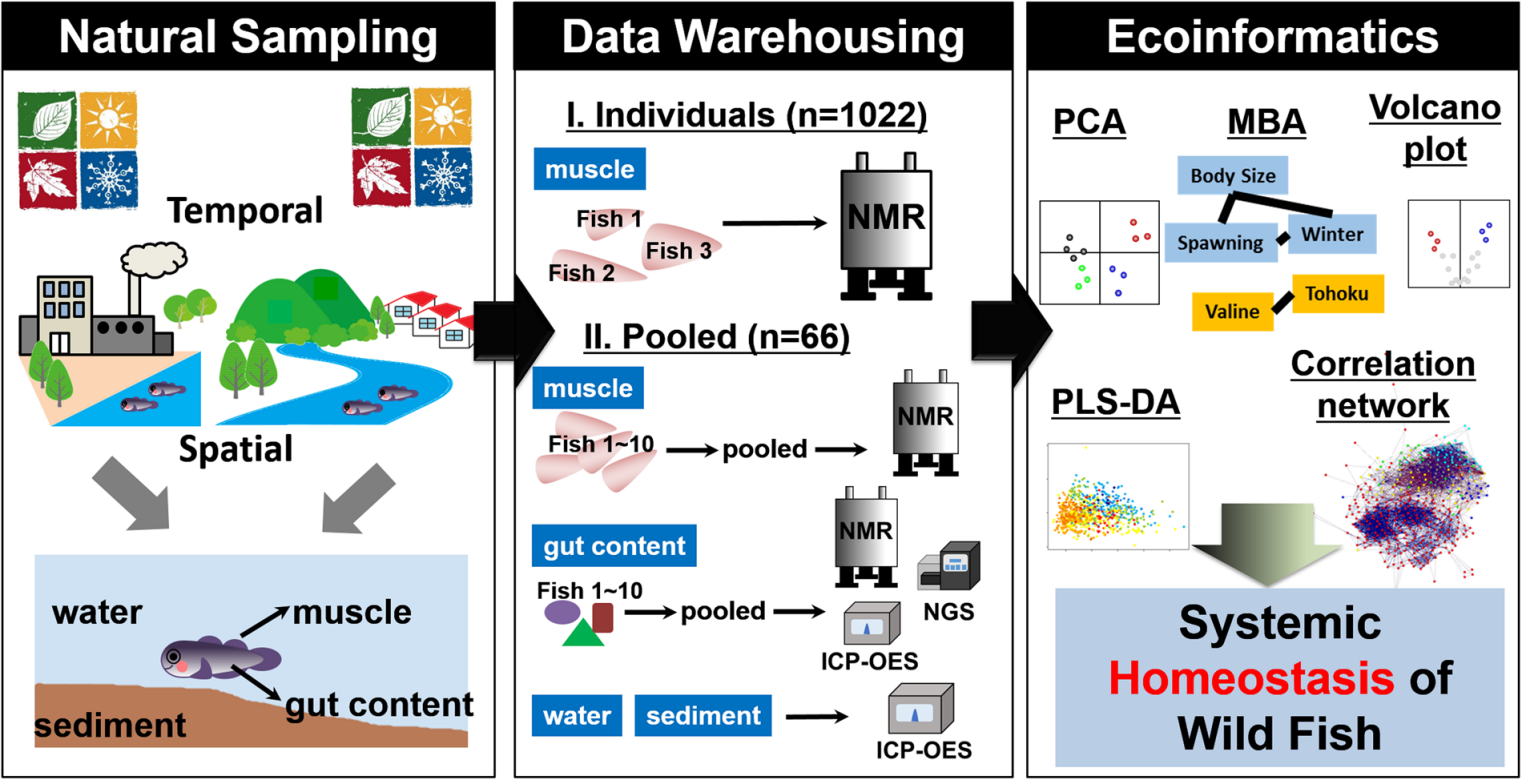
Conceptual scheme of the present study. The all figures were drawn by Feifei Wei, using R platform 3.3.3, Gephi 8.0, Adobe Illustrator CS6 and Microsoft PowerPoint 2013.

In this study, data-driven approaches were applied to investigate the temporal and spatial changes in metabolome, ionome, and microbiome of the wild yellowfin goby, and its potential interaction with the estuarine environment. The scheme of the present study is shown in Fig. 1. Over 1,000 yellowfin goby were randomly collected during three years, from May 2012 to November 2014, from the natural estuarine areas of 29 rivers throughout Japan. This data set enables the construction of multiscale models of the complex wildlife-habitat systems. Metabolic and elemental profiles of the muscle and gut contents of yellowfin goby as well as the river water and sediment samples from their habitats were measured using two-dimensional ^1^H-^1^H J-resolved (2D-J) and ^13^C NMR spectra, and inductively coupled plasma optical emission spectrometry (ICP-OES). Microbial profiles of gut microbiota were characterized using next-generation 16 S rRNA gene sequencing (NGS). Due to the technical limitations, there are two analytic strategies in this study. The first involved acquiring the metabolic profiles of each individual yellowfin goby using the cost-effective, high-performance NMR-based metabolomic analysis, aimed to investigate the metabolic responses of each individual to internal factors of growth and development. The second involved acquiring the metabolic, elemental, and microbial profiles of the pooled samples generated, according to either the same habitat or the same sampling season as well as the river water and sediment samples from their habitats, and was aimed at investigating systemic relations among wild yellowfin goby, symbiotic microorganisms, and environment from a general view. Unsupervised clustering approaches, such as principle component analysis (PCA), X-means nonhierarchical cluster analysis, and partial least squares discriminant analysis (PLS-DA) were employed to reduce the dimension of the complex big data sets. Market basket analysis (MBA) as well as correlation networks using statistical tools was performed to mine the significant factors affecting population characteristics as well as the influence of environmental factors such as temperature, salinity and inorganic elements on metabolic and microbial profiles of yellowfin goby.

## Results

### Growth stage is a primary factor affecting muscle metabolite

**Figure 2:**
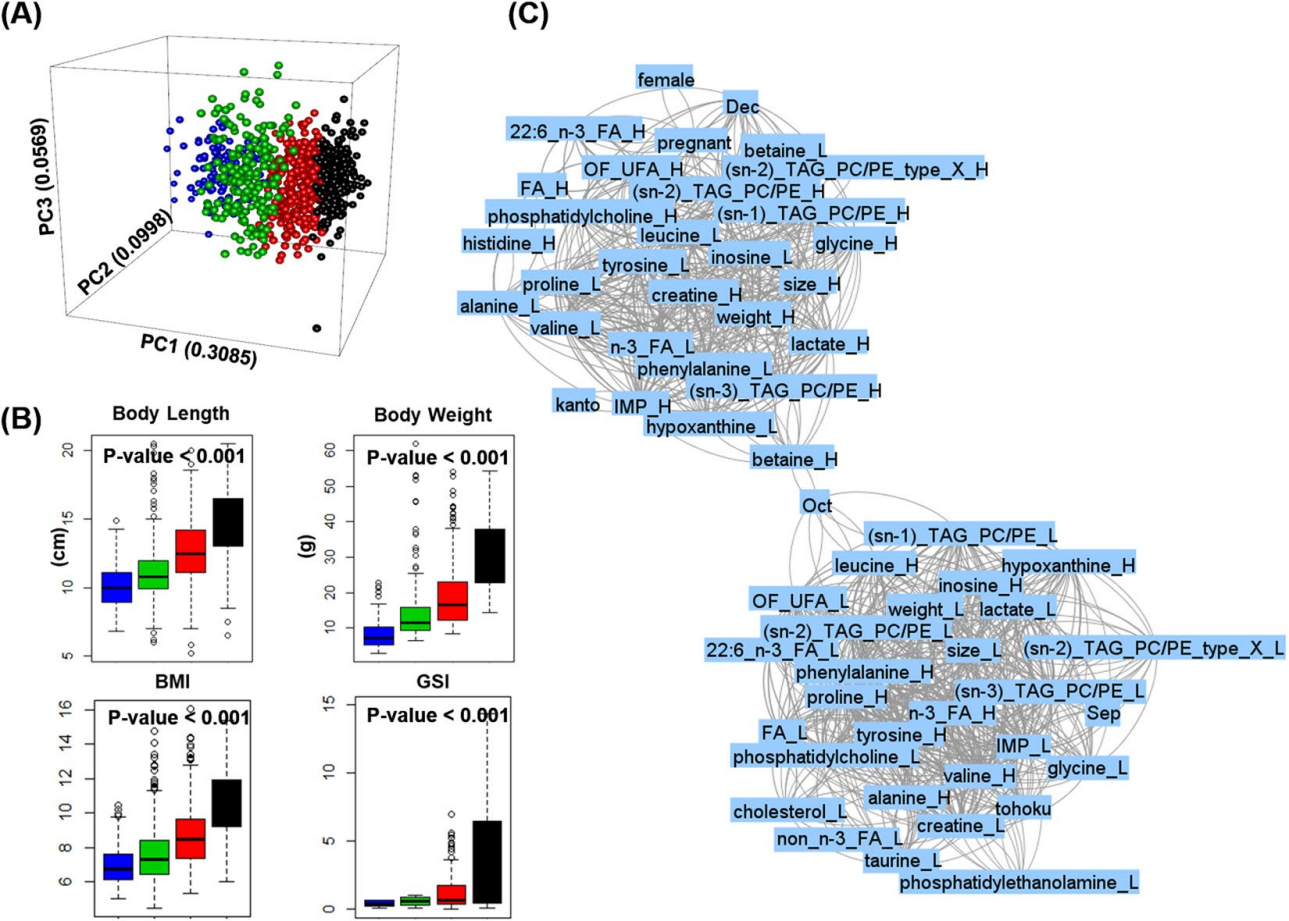
Growth stage is the primary factor affecting muscle metabolites. (**A)** PCA score plot (PCs 1~3) of all 1022 NMR spectral data of wild yellowfin goby muscle samples, colored according to the results of x-means clustering analysis; (**B**) box-plots of body length, body weight, BMI and GSI of wild yellowfin goby clustered by x-means: color is consist with clusters in (**A**). The P-values were calculated using ANOVA; **(C)** MBA network of sampling factors and muscle metabolites of wild yellowfin goby: support = 0.625, confidence = 0.25, lift >1. “_L” indicates metabolites at low level, and “_H” indicates metabolites at high level. Abbreviation of metabolites are consistent with those in Table S7.

The unsupervised PCA and x-means clustering analysis were performed on all 1,022 NMR spectra of wild yellowfin goby muscle samples. The x-means analysis is one of the most famous partitioning clustering algorithm that separate a set of instances into clusters according to their similarity. The fishes were discriminated into four clusters, where were labeled with different colors and presented in the PCA three-dimensional (3D) score plot of PC1 versus PC2 versus PC3 (Fig. 2A). As the results, the x-means exhibit similar patterns of discrimination with the PC1 (0.3085). The distributions of body length, body weight, BMI and GSI (Fig. 2B and Table S1) among the four resulting fish clusters showed a stage-dependent increasing trend. To further extract the muscle metabolites contributing to the clustering in x-means analysis, PLS-DA was applied in muscle NMR spectral data of yellowfin goby fish (Figure S1). Assignment of the NMR spectral peaks extracted from the PLS-DA loading plots indicated the difference in the content of muscle metabolites such as leucine and valine contributing to draw the lines to separate the yellowfin goby fish into 4 groups (Table S2). Further, a network of MBA is applied for identifying metabolic signatures under diverse environmental stresses in a functional context. As shown in the interaction network generated between NMR spectral data and the characteristic factors (Fig. 2C), MBA generated two separated gatherings, referred hereafter as “big-gathering” and “small-gathering.” The big-gathering contains characteristic factors including large size, heavy weight, spawn, and the mature season December as the time factor; these factors are generally highly associated with each other. In contrast, the small-gathering contains characteristic factors including small size, light weight, and the time factors September and October prior to the mature season. The habitat factor Kanto is plotted in the big-gathering exhibiting high association with large size and heavy weight, whereas the habitat factor Tokoku is plotted in the small-gathering. Notably, the big-gathering is characterized by representative muscle metabolites of unsaturated fatty acids, creatine, lactate, and IMP, whereas the small-gathering is characterized by hypoxanthine and amino acids components such as leucine, valine, alanine, proline, tyrosine, phenylalanine, and inosine.

The results in Fig. 2 indicate that NMR-based metabolic profiling of the muscle samples is characterized by the growth phases of the organism. We assume that the high amount of muscle metabolites such as creatine and lactate responds to the strong athletic ability of large individuals. Regarding branched chain amino acids (BCAA), leucine and valine tend to be fully utilized in the formation of muscle tissue in large individuals, whereas in contrast they exist in free forms in the muscle tissue of small individuals. Taken together, the MBA network provides a new perspective for comprehensively characterizing high dimensionality and large data sets.

### Fertility related muscle metabolism

**Figure 3:**
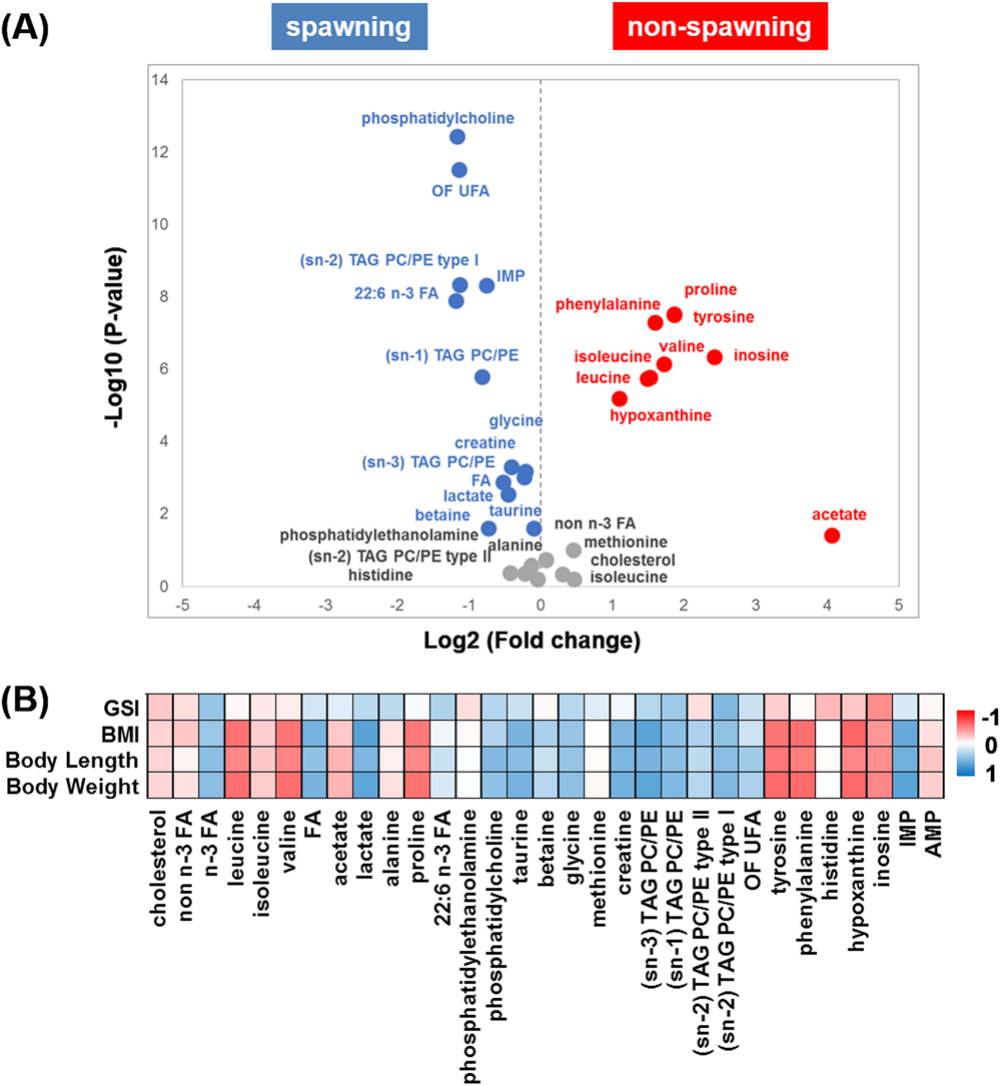
Muscle metabolites related with female yellowfin goby fertility. (**A**) Volcano plot of muscle metabolites between spawning and non-spawning female yellowfin goby: significant variables (P < 0.05 and fold changes <0.8 or >1.2) are highlighted; (**B**) Spearman’s correlation coefficients between GSI and NMR-visible metabolites of spawned female yellowfin goby. NMR peaks with high correlation coefficient (>0.4 or <−0.4) are highlighted.

The Volcano plot of the NMR spectral data of female wild yellowfin goby shown in Fig. 3A provides detailed information on muscle metabolites associated with fertility. When individuals with and without spawn are compared, high contents of fatty acids, glycerol, IMP, creatine, lactate, glycine, betaine, and taurine are detected in the muscle tissue of the spawning group, whereas high contents of phenylalanine, proline, tyrosine, valine, leucine, n-3 fatty acids, inosine, hypoxanthine, and acetate are detected in the muscle tissue of non-spawning group. This result is consistent with spawning occurring near the end stage of growth: most components increase with growth and appear also to be present in the spawning group as shown in Fig. 2C. In contrast, metabolites decreasing during growth are present in the non-spawning group. Intriguingly, as shown in Fig. 2C, taurine with low intensity is plotted in the small-gathering, whereas taurine with high intensity is absent in the big-gathering. Furthermore, significant difference in the content of taurine was observed between spawning and non-spawning groups (Fig. 3) Besides BCAA, amino acids such as proline, tyrosine and phenylalanine, which are conditionally essential, nonessential, and essential amino acids, respectively, were found to be more abundant in the muscle tissue of the non-spawning group. High intensity of acetate was also observed in the non-spawning group.

To further understand the quantitative relation between metabolites and the growth and development of wild yellowfin goby, we calculated the correlation efficiency between metabolites and body length, BMI, and GSI (Fig. 3B). Interestingly, the two representative phospholipids phosphatidylethanolamine (PE) and phosphatidylcholine (PC) did not correlate in a similar way, the content of PC was increased with body weight and BMI, whereas PE showed limited correlation with these two characterization factors but showed a weak negative correlation with GSI. This suggests that while PC is involved in the accumulation of body fat during the wild yellowfin goby growth, PE may rather be related to physiological regulation processes. In addition, alanine, a glycogenic amino acid well characterized in the gluconeogenesis pathway of many fishes^26,27^, was negatively correlated with body weight and BMI but positively correlated with the maturity indicator GSI of spawning female fish. It is known that migratory fishes that spawn once in a life time often imply a great energy investment prior to spawning and rely entirely on endogenous energy stores to fuel return migration^28^. In accordance with the observations in American shad (*Alosa sapidissim*)^29^ and chum salmon^30^, the decreases of lipid and protein contents and increase of glycogenic amino acid in yellowfin goby muscle reflect a physiological role of gluconeogenesis occurred in muscle in feeding the gonad during the long distance migration.

### Influence of latitudinal distribution on muscle metabolism

**Figure 4:**
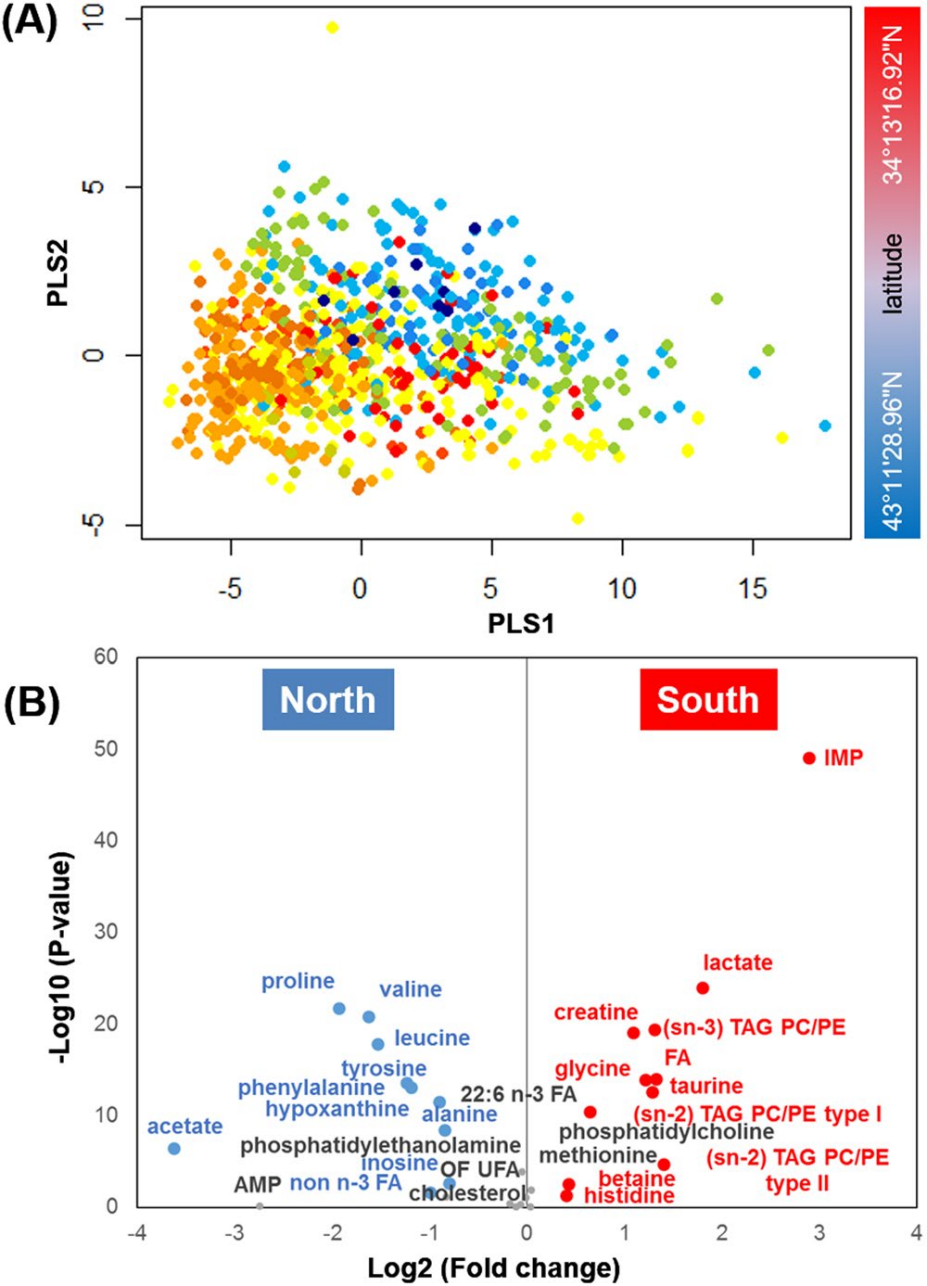
Influence of habitat latitude on muscle metabolic profiles of yellowfin goby. (**A**) PLS-DA score plot (PLSs 1 and 2) of all the 1022 NMR spectral data of wild yellowfin goby muscle samples: the North group, habitat latitude >38° N; the South group: habitat latitude <38° N. Fish samples are colored according to their habitat latitude; (**B**) Volcano plot of muscle metabolites between the North and South groups: significant variables (P-value < 0.05 and fold changes <0.8 or >1.2) are highlighted.

The MBA-generated network suggests that latitudinal distribution might contribute to the impact of habitat on metabolite composition of wild yellowfin goby muscle. The samples were thus divided into two groups according to the latitudes of their sampling location. The score scatter plot of PLS-DA showed clear latitude-dependent discrimination based on the muscle metabolite profiling data (Fig. 4A). Furthermore, the volcano plot of the NMR spectral data of the two groups revealed that the South group contains high levels of creatine, IMP, lactate, glycerol, fatty acids, betaine, histidine, and taurine, whereas the North group contains more proline, alanine, tyrosine, valine, leucine, phenylalanine, hypoxanthine, inosine, and acetate (Fig. 4B). The observed difference is likely due to the difference in water temperatures (as shown in Figure S2). Increased contents of muscle amino acids such as tyrosine and valine was observed in the North group with a relatively lower water temperature, implying that these amino acids are indeed involved in the physiological adaptation of wild fish to low water temperature. In contrast, the content of most muscle glycogenic amino acids such as alanine, proline, and tyrosine are consistent in the North group at the high-latitude region with the lack of food source. Histidine, as a physiological modulator of a broad spectrum of biological processes of fish including environmental stress response^31^, was rich in the South group (Fig. 4B) as well as in the early stage of spawning season, and decreased with increasing GSI values (Fig. 3B), which might be partly related to the functional regulation of muscle contraction and other physiological modulations.

### Systematic analysis of metabolome, ionome and microbiome of the pooled fish samples

To further investigate the relationship between living organisms and the environment, the samples collected in the same habitat were pooled together, resulting in a total of 66 pools of different habitats and sampling times as shown in Table S3. Then, the metabolic profiling of muscle tissues and intestinal contents was carried out using ^1^H and ^13^C NMR spectroscopy, and ICP-OES measurement. The intestinal microbiota was characterized by 16 S rRNA gene pyrosequencing. The water and sediment samples collected at the habitats were characterized using ICP-OES measurement. The generated high dimensionality and large data sets were preprocessed and integrated as previously described^8^. The PCA score plots of each data set are shown in Figure S3. Generally, the metabolic profiles showed a slight time-dependent distribution according to growth stage and sampling season, whereas the elemental profiles were closely correlated with geographical location and chemical composition of the habitat environment. The PCA profiles of intestinal microbiome showed limited correlation with growth stage and sampling season in the pooled samples. Furthermore, similarity score correlation analysis using the integrated datasets of the 66 pooled samples indicated that the samples obtained from the same season and geographic location showed a high level of similarity, suggesting that our integrated analytics strategy is efficient to monitor and predict the ecosystem dynamics (Figure S4).

**Figure 5:**
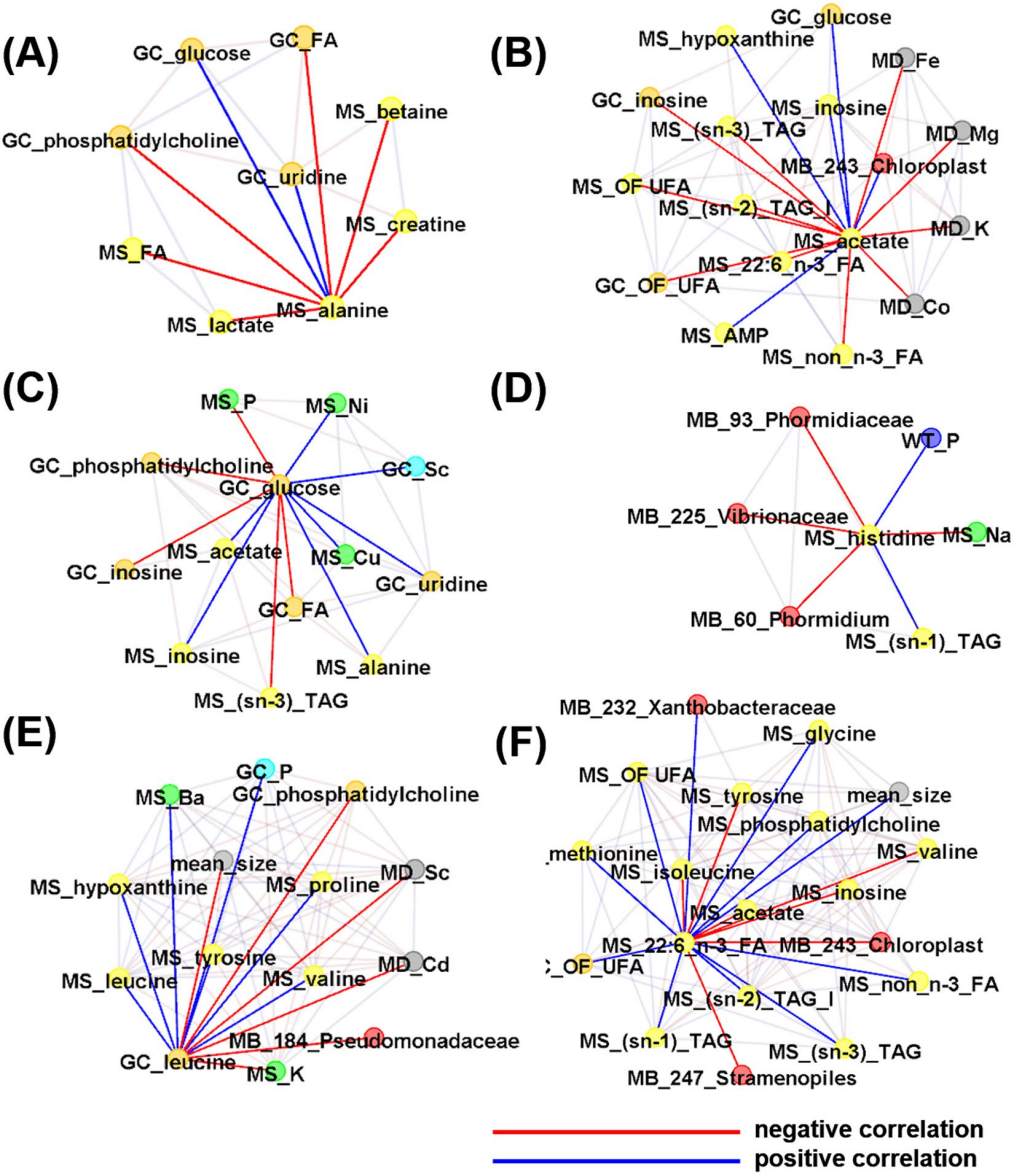
Systematic analysis of metabolome, ionome, and microbiome of the pooled fish samples. Networks related with (**A**) muscle alanine; (**B**) muscle acetate; (**C**) glucose in gut contents; (**D**) muscle histidine; (**E**) leucine in gut contents; (**F**) 22:6 n-3 fatty acid (docosahexaenoic acid, DHA). Spearman’s correlation coefficients (cor) between variants of integral data sets were calculated, and correlations with |cor| >0.5 were selected as edges. MS, muscle; WT, water; MD, sediment; GT, gut content; MB, gut microbiota. The ID code of gut microorganism is consistent with Table S8.

Then, correlation clustering and network analysis (Figure S5) were performed to investigate the hidden patterns and interactions related with energy metabolism (Fig. 5A–C), the physiological modulator histidine (Fig. 5D), and essential amino acids and fatty acids (Fig. 5E) in the integrated datasets. The content of muscle alanine was negatively correlated with intestine and/or muscle metabolites PC and fatty acids, and positively correlated with intestinal uridine and glucose. Muscle alanine was also negatively correlated with lactate, creatine, and betaine in muscle, the relative content of which reflect muscle strength and motor ability (Fig. 5A). The content of muscle acetate is negatively correlated with sediment inorganic elements such as Fe, Mg, K and Co, muscle unsaturated n:3 fatty acids, intestinal unsaturated fatty acids and inosine, and is positively correlated with energy-related muscle metabolites such as AMP, inosine and hypoxanthine, intestinal glucose, and Cyanobacteria CAB.I (Fig. 5B). In accordance with the above observations, intestinal glucose is positively correlated with muscle alanine and acetate as well as intestinal scandium (Fig. 5C). Muscle histidine is negatively correlated with muscle Sodium ion (Na) and intestinal microbiota Cyanobacteria Phormidiaceae Proteobacteria Vibrionaceae and positively correlated with muscle glycerol and inorganic phosphorus (P) in environmental water, indicating its physiological role in the regulation of muscle osmotic pressure (Fig. 5D). The essential amino acid leucine is positively correlated in muscle and intestine of wild yellowfin goby (Fig. 5E). This is consistent with its dietary origin. Furthermore, intestinal leucine was found to be negatively correlated with intestinal Proteobacteria Pseudomonadaceae (Fig. 5E). The muscle essential fatty acid 22:6 n-3 fatty acid (DHA) is positively correlated with mean body size of pooled fish, muscle lipid components and intestinal unsaturated fatty acids and Proteobacteria Xanthobacteraceae, and negatively correlated with muscle free essential amino acids BCAAs including valine, leucine and isoleucine, and intestinal Cyanobacteria including Cyanobacteria Stramenopiles and Cyanobacteria CAB.I (Fig. 5F).

### Influence of seasonal variation in regulating yellowfin goby body homeostasis

**Figure 6:**
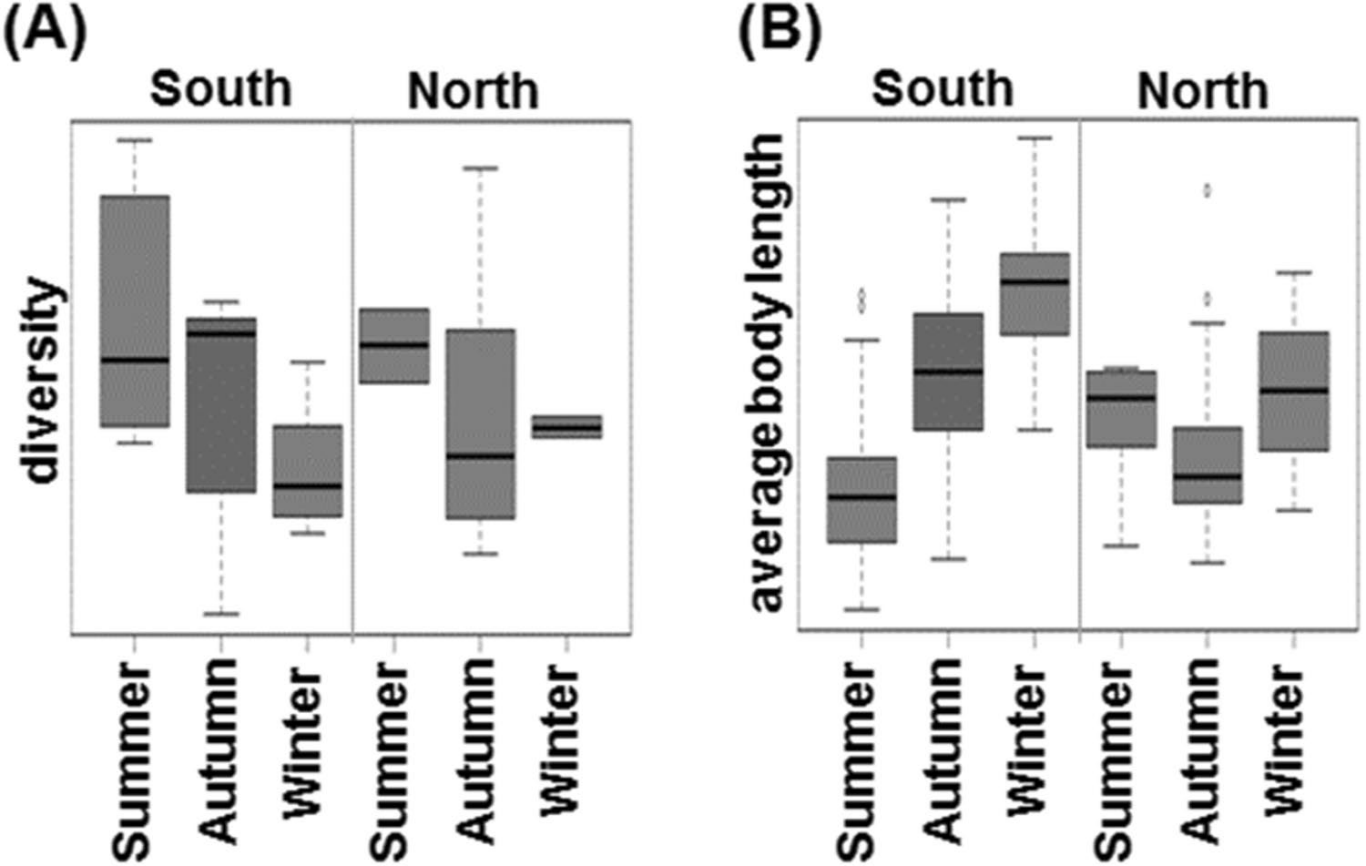
Influence of seasonal variation in regulating the body homeostasis of yellowfin goby. Boxplots of (**A**) diversity of gut microbiota and (**B**) average body length of yellowfin goby collected in the South and North regions in different seasons.

The seasonal variation in the intestinal microbial diversity and average body length in the South and North groups are shown in Fig. 6. In the two groups, seasonal variation in the intestinal microbial diversity (*p*-values of South, 0.08; North, 0.81) of yellowfin goby tended to decrease from summer to winter. Intriguingly, the average body length of the southern annual yellowfin goby increased continuously, whereas the northern biennial yellowfin goby showed absence of average body length difference over the year. In addition, increased intestinal microbial diversity is observed in the South group, compared with that in the North group. Previous ecological study in Japan demonstrated that the lifespan of yellowfin goby is closely related with its surrounding environment, especially the temperature. In 1940s, the ratio of annual and biennial fishes in Tokyo Bay in Kanto is approximately 1:1^32^. The major population of yellowfin goby in Ibaraki region, where is located between Kanto and Tohoku, changed from biennial fish in 1970s to annual fish in 1990s, whereas most of yellowfin goby is still biennial fish until 1993 in the northernmost region of Japan^33^. Therefore, the seasonal variation in intestinal microbial diversity and average body length observed in the South group but not the North group is likely due to a mixed population of annual and biennial fishes as well as their difference in migration pattern in the North group. Besides temperature, during the migration between freshwater and seawater, the symbiotic intestinal microbiota of yellowfin goby can also be influenced by other environmental factors such as water salinity (Figure S6)^34^.

## Discussion

This data-driven study is initiated to establish an integrated model and mine the temporal and spatial interactions in metabolome, ionome, and microbiome of the wild yellowfin goby and the estuarine environment. Our findings show that the chemical composition of living organisms is tightly affected by their environment, which in turn suggests that a chemical composition analysis can be used to predict habitats of living organisms. Size is the defining characteristic of big data. With the rapidly accumulating large-scale molecular-biological data, pattern mining and correlation clustering of big data sets provide powerful and information-rich approaches to understand the dynamic network of interactions and thereby extract critical features of complex ecosystems in response to either natural or artificial environmental stimuli. In contrast, this information is difficult to obtain using single-condition controlled experimental models. Although big data are often noisy due to complexity itself, it is still valuable for discovering previously unknown and potentially useful hidden patterns.

An intriguing finding is the correlation between muscle taurine content and the spawning of yellowfin goby. MBA has been proven as a powerful approach in omics studies to investigate the relations of phenotypes and biomarkers as an alternative to traditional single-metabolites analysis^9,35–37^. Spawning is plotted in the big-gathering in the MBA-generated network generated between the muscle NMR spectral data of yellowfin goby and their characteristic factors, suggesting that fertility is a physiological phenomenon occurring after maturation. Further, low content in the muscle of young yellowfin goby but is not consistently high in adult individuals and a significant difference between the spawning and non-spawning groups pointed out a critical role of taurine in the control of the reproductive function of wild female yellowfin goby through the regulation of spawning. This result is consistent with previous studies on the relation between improved reproductive performance and dietary taurine in other farmed fish^38,39^. Besides, taurine is involved in various important physiological functions such as bile salt synthesis, osomoregulation, and modulation of neurotransmitters, membrane stabilization and antioxidation in mammals^40^. It is possible that increased content of muscle taurine might relate to the improved skin condition which results to a decrease in scale detachment and fish mortality during land transportation^41^. Similar to the significant difference between the spawning and non-spawning groups, the content of taurine was also significantly different between the low and high latitude areas. Most yellowfin goby in Japan are annual fish, whereas they are mostly biennial in high latitude areas such as the Tohoku region. The slow maturation of yellowfin goby at high latitude is likely to be related with the low water temperature, which will reduce the food intake of fish and lead to their starvation^42^. This may also explain the low content of taurine due to the surrounding environment (such as the lower temperature), which may further relate to a delayed maturation of high-latitude fish.

Our systematic analysis of metabolome, ionome and microbiome of the pooled fish samples uncovered the hidden patterns and novel interactions in the integrated datasets with specific focus on energy metabolism, the physiological modulator histidine, and essential amino acids and fatty acids. An important difference in energy metabolism between wild fish and aquaculture fish is the activation of gluconeogenesis due to food insufficiency. Our data revealed that the content of muscle alanine and acetate were negatively correlated with body length, body weight and BMI, and positively correlated with GSI, suggesting alanine and acetate are likely the principal substrates for energy metabolism due to the lack of adequate food resources, especially in the case of insufficient fat source. From a long-term point of view, abundance of food sources, especially a rich source of meat such as fish and shrimp in the low-latitude region, would certainly provide good nutrition for the growth of wild fishes. It is well known that the primary energy source for carnivorous and omnivorous fish is not sugar but protein^43^. However, the yellowfin goby will also eat plants and algae in the case of insufficient protein source. During prolonged fasting, acetate generated by the *β*-oxidation of fatty acids has been shown to serve as the energy source for muscle in the rat^44–46^. Increased muscle acetate observed in the non-spawning group and the North group at the high-latitude region suggests that fatty acid *β*-oxidation is also an important process for yellowfin goby to produce energy under scarce food conditions. These results suggest that muscle acetate is involved in energy metabolism, and is probably produced from unsaturated n:3 fatty acids oxidation in the muscle. Since both muscle alanine and acetate are strongly correlated with intestinal glucose, we propose that intestinal glucose could predict the food consumption status of wild yellowfin goby. In addition, intestinal glucose is also positively correlated with intestinal scandium. As an estuary heavy metal iron resident in sediment, scandium has been reported to affect the health of bottom-dwelling fishes^47^. The yellowfin goby is a coastal demersal fish and will likely swallow sediment containing scandium. This data indicates that the metabolic status of yellowfin goby is tightly influenced by environmental stress such as food availability.

Histidine is an important physiological modulator involved in the thermoregulation in various fishes^31^. Different to other muscle metabolites, histidine is associated with neither body weight nor BMI, suggesting it is a relatively stable physiological regulator during wild yellowfin goby growth. Importantly, it is negatively correlated with GSI, implying an involvement in the reproductive system regulation, especially in the early stage of spawning. Indeed, increased muscle histidine is observed in salmon prior to the spawning migration^48^. Intriguingly, muscle histidine is negatively correlated with intestinal microbiota Cyanobacteria Phormidiaceae and *Phormidium sp*., which is a phylum of photosynthetic bacteria, ubiquitous in aquatic habitats, especially in freshwater habitats. The reproduction of yellowfin goby is known to not occur in low salinity estuarine areas. Pelagic larvae of yellowfin goby use tidal currents to migrate upstream towards upper freshwater habitats to complete their maturation in autumn and winter, and subsequently migrate downstream to areas of higher salinity to spawn during the next spring^49^. It is possible that the content of intestinal Cyanobacteria reflects the spawning migration of yellowfin goby.

Furthermore, we investigated networks related to essential amino acids and essential fatty acids that cannot be synthesized by the organism but must be provided by the diet. Intriguingly, the essential amino acid leucine and essential fatty acid DHA are correlated with intestinal Proteobacteria Pseudomonadaceae and Proteobacteria Xanthobacteraceae, respectively. These results highlighted the contribution of intestinal bacteria to the host nutrition. Our data suggests that family Pseudomonadaceae, which has received a great deal of attention because it contains a BCAA-degrading enzyme^50–52^, is involved in the degradation of BCAA in wild yellowfin goby. Muscle DHA in fish can be derived from food such as small fishes or synthesized from the intestinal microbial metabolism^53^. Our data indicates that the content of muscle DHA increases with yellowfin goby growth. Further studies are needed to elucidate the role of intestinal Proteobacteria Xanthobacteraceae in the synthesis of DHA in fish.

Besides temperature, water salinity is another important environmental factor that influence the symbiotic intestinal microbiota of yellowfin goby during their migration between freshwater and seawater. Yellowfin goby is well known as a euryhaline fish that live mostly in salt water but are capable of living under a wide range of salinities. Although yellowfin goby might survive in fresh water for couple of months, they require saline water (salinities greater than 5 ppt) for successful reproduction and most of them will die in fresh water during the spawning period^54^. Further study is needed to uncover the physiological role of osmoregulatory and metabolic changes in gills, kidney, liver and brain^55^ in maintaining the homeostasis of yellowfin goby during spawning migration.

In summary, seasonal and latitudinal changes on the metabolome, ionome and microbiome of wild yellowfin goby were observed and their potential interaction with the estuarine environment such as temperature might play an important role in body homeostasis of wild yellowfin goby. In a previous study^56^, NMR-based metabolic analysis of gilthead sea bream under non-isothermal conditions revealed that muscle lipid production such as cholesterol, phosphocholine, and fatty acids provided energy source for the ectothermic fish in response to sudden ambient temperature change. Our results further demonstrate that symbiotic intestinal microbiota is more sensitive than their host to the environment, such as the temperature. These findings are supported by a previous meta-analysis showing that environmental and ecological factors shape the gut bacterial communities of fish^57^. These data suggest that environment rather than the host growth stage influence the change in intestinal microbial diversity from summer to winter. Therefore, we conclude that the environmental factors such as the temperature plays important roles in maintaining the sustainable living system of wild fish, which should be taken into consideration to systematically evaluate the impact of global climate change on the ecosystem.

## Methods

### Sample collection and preprocessing

Yellowfin gobies (n = 1,022) were collected over a period of three years from May 2011 to November 2014 from the estuarine and coastal zones of 29 rivers in Kanto, Tohoku, Hokkaido, Chubu, Kinki and Chugoku regions of Japan as shown in Table S4. There is no specific permission required for all of the sampling points as they are all public places. The animal experiments were performed in accordance with protocols approved by the Institutional Committee of Animal Experiment of RIKEN and adhered to the guidelines in the Institutional Regulation for Animal Experiments and Fundamental Guidelines for Proper Conduct of Animal Experiment and Related Activities in Academic Research Institutions under the jurisdiction of the Ministry of Education, Culture, Sports, Science and Technology, Japan.

Water and sediment from each habitat were collected as a set. After measuring the body length and weight, the fish samples were dissected, their sex identified, their gonadal status/maturation checked, and gutted. The fish was defined as spawning or non-spawning according to the enlargement of gonads and presence of eggs in the ovary at this time. Body mass index (BMI) and gonadosomatic index (GSI) were calculated using the following formulae:

1 $$BMI=\frac{BW\times 100}{S{L}^{2}}$$

2 $$GSI=\frac{GW\times 100}{BW}$$

where GW stands for gonadal weight, BW for body weight, and SL for standard length.

Body muscles and gut contents were collected, lyophilized and crushed into powder for chemical and biological analysis.

### NMR observation of individual fish samples

For NMR observations, 30 mg of each powdered sample was extracted with 1000 mL of deuterated methanol (99.8%, Cambridge Isotope Laboratories Inc., MA, USA) with 1 mM sodium 2,2-dimethyl-2-silapentane-5-sulfonate (DSS) internal standard at 55 °C for 15 min. After centrifugation at 25 °C for 5 min, the extracted supernatant was transferred to a 5 mm NMR tube. Two dimensional J-resolved (2DJ) spectra were acquired at 298 K using a Bruker AVANCE II 700 spectrometer equipped with a ^1^H inverse triple-resonance cryogenically cooled probe with Z-axis gradients (Bruker BioSpin GmbH, Rheinstetten, Germany).

### Pooled fish samples for integral analysis

For integral analysis, the fish powder was pooled from ~10 individuals sampled at the same season and habitat as shown in Table S3. The pooled powder samples, both body muscle and gut contents, were labeled by their sampling time and location, and processed for ^1^H and ^13^C NMR, ICP-OES analyzes. Community analysis of enteric microbiota was performed only on gut content powders to retrieve information on normal endogenous microbiota. Water and sediment samples were analyzed using ICP-OES.

### ICP-OES of pooled fish samples

Fish powder samples were incubated with 6 mL of aqueous nitric acid (6.9% v/v) in a Thermomixer comfort (Eppendorf Japan, Tokyo, Japan). Collected supernatants were filtered through a Millex GS filter (0.22 mm, EMD Millipore, Billerica, MA) using SPS5510 (Hitachi High-Tech Science Corporation, Tokyo, Japan). The sediment samples were freeze-dried, and 10 mg of the powder was incubated with 1 mL of methanol at 50 °C for 15 min. After centrifugation (14000 rpm, 5 min), the supernatants were removed and 2 mL of aqueous nitric acid (6.9% v/v) was added, and the sample incubated at 50 °C for 15 min. The supernatants were collected after centrifugation (14000 rpm, 5 min), and the extraction using 2 mL of aqueous nitric acid (6.9% v/v) was repeated three times. The water samples were diluted to ten times using milli-Q water, and then used for ICP-OES analyzes. The ICP-OES analysis was conducted using an SPS5510 instrument, with a range of wavelengths from 167 to 785 nm and 74 applicable elements. The ICP-OES data is available in Table S5.

### ^1^H and ^13^C NMR observations of pooled fish samples

Pooled fish powder samples underwent both ^1^H and ^13^C NMR observations. The ^1^H NMR spectra were detected the same way as described above, and the ^13^C NMR spectra were detected at 125 MHz at 298 K using a Bruker AVANCE II 500 spectrometer (Bruker BioSpin GmbH, Rheinstetten, Germany). The parameters were as follows: data points, 64 K; number of scans, 3584; spectral width, 29762 Hz; acquisition time, 1.10 s.

### Microbial community analysis of pooled fish samples

Microbial DNA extraction was performed according to previous studies with slight modifications. Each DNA sample was amplified by PCR using universal bacterial primers 954 f and 1369r targeted to the V6–V8 regions of the 16 S rRNA gene according to a previous report. The sequencing analysis and the data processing were outsourced to Operon Biotechnologies Co. Ltd. (Tokyo, Japan). Categorization of bacterial taxa was performed using a Ribosomal Database Project (RDP; http://rdp.cme.msu.edu/seqmatch/seqmatch intro.jsp) classifier^58^. The microbial profile data is available in Table S6.

### Data processing and statistics analysis

All 1D ^1^H-NMR data were baseline collected and peak picked by rNMR^59^ on the platform of “R.” Each spectrum was normalized by PQN^60^, scaled, centered and then sorted out as basic data matrix. Statistical multivariate analyzes, such as principal component analysis (PCA), X-means nonhierarchical cluster analysis, partial least squares discriminant analysis (PLS-DA), volcano plot, market basket analysis (MBA)^9^ were performed using R to meet the requirements of data mining. The network diagram was rendered in Gephi (http:// gephi.org) according to the previous studies^61^.

For the pooled fish samples, the integral data set includes ^1^H NMR/^13^C NMR spectral data of body muscle and gut contents, ICP-OES data of body muscle, gut contents, habitat water and sediment, as well as the MiSeq data of gut contents. PCA, PLS-DA, Spearman’s relation was performed by R software with a package “Muma.”

### NMR signal annotation

NMR signals were annotated using 2D SENSI^11^, STOCSY^62^, SHY^63^ and other observed 2D NMR spectra as reported previously^15,17^. Detailed signal annotation is summarized in Table S7, Figures S7 and S8.

## Electronic supplementary material

Supporting information

Table S4

Table S5

Table S6
