# Supplementary material for: Systemic Homeostasis in Metabolome, Ionome, and Microbiome of Wild Yellowfin Goby in Estuarine Ecosystem
Source: Sci Rep. 2018 Feb 22;8:3478. doi: 10.1038/s41598-018-20120-x (PMC5823927; doi:10.1038/s41598-018-20120-x)
Supplement: Supplementary file 1 — Supporting information [file 41598_2018_20120_MOESM1_ESM.pdf]

SUPPORTING INFORMATION for:

Systemic Homeostasis in Metabolome, Ionome and Microbiome of Wild Yellowfin Goby in Estuarine Ecosystem

Feifei Wei[1], Kenji Sakata[1], Taiga Asakura[1, 2], Yasuhiro Date[1, 2], and Jun Kikuchi[*, 1, 2, 3]

[1]RIKEN Center for Sustainable Resource Science, 1-7-22 Suehiro-cho, Tsurumi-ku, Yokohama 235-0045, Japan

[2]Graduate School of Medical Life Science, Yokohama City University, 1-7-29 Suehirocho, Tsurumi-ku, Yokohama 230-0045, Japan

[3]Graduate School of Bioagricultural Sciences and School of Agricultural Sciences, Nagoya University, 1 Furo-cho, Chikusa-ku, Nagoya 464-8601, Japan

Contents:

Table S1. Distribution of body length, body weight, BMI and GSI in the groups clustered using x-means.

Table S2. Top muscle NMR spectral peaks extracted from PLS-DA loading plots.

Table S3. Sample list of pooled yellowfin goby in the present study.

Table S4. Sample list of yellowfin goby in the present study. (Attached as an Excel file)

Table S5. ICP-OES datasets of muscle, gut contents, river water and sediment of the yellowfin goby living systems. (Attached as an Excel file)

Table S6. Intestinal microbial profiles (family level) of yellowfin goby. (Attached as an Excel file)

Table S7. NMR signal annotation of yellowfin goby muscle extracts using Methanol-*d4*.

Table S8. Microorganism name list highlighted in the network analysis.

Figure S1. PLS-DA analysis of muscle NMR spectral data of yellowfin goby fish. **(A)** score plot; **(B)** loading plots of PLS1 and PLS2. The top 30 NMR peaks in the loading plots were assigned and summarized in Table S2. The color of groups was consistent with x-means clusters in Figures 2 and Tables S1.

Figure S2. The average temperature of Sendai City and Yokohama City from years 2011 to 2013.

Figure S3. PCA score plots (PC1 vs. PC2) of NMR-based metabolic profiling, ICP-OES-based elemental profiling and Miseq-based microbial profiling of the living system of pooled yellowfin goby.

Figure S4. The similarity score of yellowfin goby ecosystems.

Figure S5. Correlation network analysis of metabolome, inonome and microbiome of the pooled yellowfin goby samples.

Figure S6. MBA network analyses of integral data sets obtained by pooled yellowfin goby, which partial highlighted by salinity with internal microbiota.

Figure S7. **(A)** ^{1}H-^{13}C HSQC spectrum and **(B)** ~ **(E)** expansions of yellowfin goby muscle extracts using methanol-d_4.

Figure S8. NMR signal annotation of lipids in yellowfin goby muscle extracts using methanol-d_4. Distinguishable signals between **(A)** phosphatidylethanolamine and phosphatidylcholine; **(B)** n-3 fatty acids and non n-3 fatty acids; **(C)** 22:6 n-3 fatty acid and non 22:6 n-3 fatty acids.

Table S1. Distribution of body length, body weight, BMI and GSI in the groups clustered using x-means.

x-means cluster	body length		body weight		BMI		GSI	
	top1/4 (>14.7cm) %	bottom1/4 (<10.5cm) %	top1/4 (>23.6g) %	bottom1/4 (<8.4g) %	top1/4 (>10.20) %	bottom1/4 (<7.06) %	top1/4 (>2.60) %	bottom1/4 (<0.39) %
**black**	15.69	2.28	16.43	0.82	15.06	0.93	4.05	2.18
**red**	6.75	5.46	6.94	4.59	7.37	5.92	1.97	2.49
**green**	1.89	9.73	1.73	11.33	2.18	11.11	0.10	1.14
**blue**	0.20	6.16	0.00	7.65	0.31	6.85	0.00	0.31

Table S2. Top muscle NMR spectral peaks extracted from PLS-DA loading plots.

δ_H (ppm)	PLS1	PLS2	assignment
0.98	-0.1910	0.1731	leucine
1.05	-0.1860	0.1680	valine
1.27	0.0501	-0.1186	FA
1.30	0.1396	0.1093	lactate
1.44	-0.0602	0.1661	alanine
1.60	-0.1708	0.1981	cholesterol, non 22:6 FA
2.10	-0.1608	0.1589	proline
3.01	0.1214	-0.0225	creatine
3.30	0.0320	-0.0986	taurine
3.56	-0.0611	0.1709	alanine
3.84	0.1241	-0.0814	creatine
4.12	-0.1100	-0.1967	inosine
4.21	0.1725	0.0536	IMP
4.31	-0.0975	-0.1595	inosine
4.39	0.1682	0.0702	IMP
4.41	-0.0296	-0.1170	(sn-1) TAG PC/PE
4.61	-0.1214	-0.2049	inosine
4.69	0.1619	0.0650	IMP
6.00	-0.1153	-0.1930	inosine
6.07	0.1729	0.0629	IMP
6.75	-0.1552	0.1373	tyrosine
7.11	-0.1723	0.1405	tyrosine
7.30	-0.1719	0.1665	phenylalanine
7.31	-0.1814	0.1655	phenylalanine
7.33	-0.1839	0.1615	phenylalanine
7.35	-0.1554	0.1847	phenylalanine
8.04	0.1573	0.0623	IMP
8.05	-0.0960	-0.1604	inosine
8.32	-0.1109	-0.1918	inosine
8.47	0.1682	0.0482	IMP

Table S3. Sample list of pooled yellowfin goby in the present study.

Group Number[a]	Year	Season[b]	River/Harbor	Location
1	2011	Autumn	Tsurumi River	35°28'41.35"N, 139°40'59.71"E
2	2011	winter	Tsurumi River	35°28'41.35"N, 139°40'59.71"E
3	2012	summer	Tsurumi River	35°28'41.35"N, 139°40'59.71"E
4	2012	Autumn	Tsurumi River	35°28'41.35"N, 139°40'59.71"E
5	2012	winter	Tsurumi River	35°28'41.35"N, 139°40'59.71"E
6	2013	summer	Tsurumi River	35°28'41.35"N, 139°40'59.71"E
7	2013	Autumn	Tsurumi River	35°28'41.35"N, 139°40'59.71"E
8	2013	winter	Tsurumi River	35°28'41.35"N, 139°40'59.71"E
9	2014	summer	Tsurumi River	35°28'41.35"N, 139°40'59.71"E
10	2014	Autumn	Tsurumi River	35°28'41.35"N, 139°40'59.71"E
11	2011	summer	Tama River	35°31'37.91"N, 139°47'26.86"E
12	2011	Autumn	Tama River	35°31'37.91"N, 139°47'26.86"E
13	2011	winter	Tama River	35°31'37.91"N, 139°47'26.86"E
14	2012	summer	Tama River	35°31'37.91"N, 139°47'26.86"E
15	2012	Autumn	Tama River	35°31'37.91"N, 139°47'26.86"E
16	2012	Autumn	Tama River	35°31'37.91"N, 139°47'26.86"E
17	2013	summer	Tama River	35°31'37.91"N, 139°47'26.86"E
18	2013	Autumn	Tama River	35°31'37.91"N, 139°47'26.86"E
19	2013	winter	Tama River	35°31'37.91"N, 139°47'26.86"E
20	2014	summer	Tama River	35°31'37.91"N, 139°47'26.86"E
21	2011	Autumn	Abukuma River	38° 2'55.00"N, 140°55'19.00"E
22	2011	winter	Abukuma River	38° 2'55.00"N, 140°55'19.00"E
23	2011	winter	Abukuma River	38° 2'55.00"N, 140°55'19.00"E
24	2012	Autumn	Abukuma River	38° 2'55.00"N, 140°55'19.00"E
25	2012	winter	Abukuma River	38° 2'55.00"N, 140°55'19.00"E
26	2013	summer	Abukuma River	38° 2'55.00"N, 140°55'19.00"E
27	2013	Autumn	Abukuma River	38° 2'55.00"N, 140°55'19.00"E
28	2014	Autumn	Abukuma River	38° 2'55.00"N, 140°55'19.00"E
29	2011	Autumn	Natori River	38°10'34.55"N, 140°57'24.33"E
30	2011	winter	Natori River	38°10'34.55"N, 140°57'24.33"E
31	2011	winter	Natori River	38°10'34.55"N, 140°57'24.33"E
32	2012	Autumn	Natori River	38°10'34.55"N, 140°57'24.33"E

33	2012	winter	Natori River	38°10'34.55"N, 140°57'24.33"E
34	2012	winter	Natori River	38°10'34.55"N, 140°57'24.33"E
35	2013	Autumn	Natori River	38°10'34.55"N, 140°57'24.33"E
36	2011	summer	Sagami River	35°18'53.52"N, 139°22'5.42"E
37	2011	Autumn	Sagami River	35°18'53.52"N, 139°22'5.42"E
38	2011	winter	Sagami River	35°18'53.52"N, 139°22'5.42"E
39	2012	summer	Sagami River	35°18'53.52"N, 139°22'5.42"E
40	2012	Autumn	Sagami River	35°18'53.52"N, 139°22'5.42"E
41	2013	Autumn	Sagami River	35°18'53.52"N, 139°22'5.42"E
42	2013	winter	Sagami River	35°18'53.52"N, 139°22'5.42"E
43	mixed	mixed	Keihin-Unga River	35°37'51.61"N, 139°45'11.72"E
44	mixed	mixed	Horiwari River	35°25'3"N, 139°37'35.9"E
45	mixed	mixed	Yokohama Harbor	35°27'2.12"N139°39'56.41"E
46	mixed	mixed	Arakawa River	35°38'49.08"N, 139°50'44.16"E
47	mixed	mixed	Kyu-nakagawa River	35°42'2.09"N139°50'26.01"E
48	mixed	mixed	Tatumi-Unga River	35°38'44.77"N139°48'23.40"E
49	mixed	mixed	Yokojikken River	35°40'56.72"N139°49'13.87"E
50	mixed	mixed	Teizan-Unga River	38°18'4.16"N, 141° 2'10.91"E
51	mixed	mixed	Shiogama Harbor	38°19'25.09"N141° 2'53.96"E
52	mixed	mixed	Shiogama Harbor	38°19'25.09"N141° 2'53.96"E
53	mixed	mixed	Yougai Harbor	38°17'18.03"N, 141° 4'15.94"E
54	mixed	mixed	Muku River	34°41'13.11"N, 135°22'2.00"E
55	mixed	mixed	Yodo River	34°40'55.42"N, 135°25'2.98"E
56	mixed	mixed	Ibi River	35°13'18.64"N, 136°37'0.31"E
57	mixed	mixed	Ota river	34°21'38.68"N, 132°24'21.58"E
58	mixed	mixed	Kiso River	35°14'2.94"N, 136°51'37.99"E
59	mixed	mixed	Kyu-ota River	34°21'10.48"N, 132°26'11.74"E
60	mixed	mixed	Syonai River	35° 4'56.32"N, 136°50'28.99"E
61	mixed	mixed	Nagara River	35° 1'36.57"N, 136°43'3.70"E
62	mixed	mixed	Iioka Harbor	35°41'31.39"N140°43'28.29"E
63	mixed	mixed	Kurose River	34°13'40.04"N, 132°36'17.11"E
64	mixed	mixed	Saigawa River	36°36'4.58"N, 136°35'10.66"E
65	mixed	mixed	Otaru Harbor	43°11'28.96"N, 141° 1'7.60"E
66	mixed	mixed	Kinokawa River	34°13'16.92"N, 135° 8'13.08"E

[a] The group number is consistent with that in **Tables S5** and **S6**.

[b] Season was defined as: May~Sep, summer; Oct~Nov, autumn; Dec~Jan, winter.

Table S7. NMR signal annotation of yellowfin goby muscle extracts using Methanol-*d4*.

Peak Number[a]	δ_H (ppm)	δ_C (ppm)	Annotation
1	1.85	33.3	cholesterol
2	6.74	118.1	tyrosine
3	1.01	19.2	valine
4	8.49	142.3	AMP
5	1.16	26.5	cholesterol
6	0.98	24.8	leucine
7	8.45	142.3	IMP
8	3.36	48.5	proline
9	0.96	23.3	leucine
10	3.69	56.8	methionine
11	3.01	40.2	phenylalanine
12	3.72	56.8	methionine
13	3.98	58.5	creatinine
14	1.05	20.7	valine
15	7.99	147.7	hypoxanthine
16	3.58	71.9	tyrosine
17	1.46	33.9	cholesterol
18	3.47	70.6	acetylcholine
19	4.42	65.1	(sn-1) TAG type I
20	5.16	74.7	(sn-2) TAG type II
21	8.31	142.6	inosine
22	1.01	38.9	cholesterol
23	1.67	27.4	non 22:6 FA
24	4.17	65.3	(sn-1) TAG
25	8.36	142.5	AMP
26	7.62	138.2	histidine
27	1.00	59.7	cholesterol
28	8.05	148.3	inosine
29	1.76	33.9	cholesterol
30	1.52	30.6	cholesterol
31	2.39	36.5	22:6 n-3 FA
32	1.05	40.0	cholesterol

33	1.85	30.8	cholesterol
34	8.11	143.5	hypoxanthine
35	4.03	64.7	phosphatidylethanolamine
36	3.01	31.2	histidine
37	1.11	59.1	cholesterol
38	1.13	42.1	cholesterol
39	3.21	31.2	histidine
40	0.93	53.2	cholesterol
41	4.69	77.7	IMP
42	5.36	134.2	OF UFA
43	1.52	23.7	cholesterol
44	5.22	73.3	(sn-2) TAG
45	4.39	74.1	IMP
46	6.07	90.7	IMP
47	3.02	32.4	creatinine
48	0.98	16.1	n-3 FA
49	3.13	43.1	phosphatidylethanolamine
50	4.21	87.7	IMP
51	2.20	44.4	cholesterol
52	5.33	124.0	cholesterol
53	1.38	38.7	cholesterol
54	1.59	27.6	cholesterol, non 22:6 FA
55	4.07	67.3	AMP
56	3.77	57.9	histidine
57	2.32	36.5	non 22:6 n-3 FA
58	0.71	13.8	cholesterol
59	3.98	66.4	(sn-3) TAG
60	4.61	77.7	inosine
61	4.12	89.0	inosine
62	3.57	53.2	alanine
63	4.31	73.6	inosine
64	6.00	92.1	inosine
65	1.30	25.2	non n-3 FA
66	1.01	21.4	cholesterol
67	0.93	20.7	cholesterol
68	5.33	132.3	OF UFA

69	1.44	18.9	alanine
70	0.86	16.0	non n-3 FA
71	1.27	34.6	FA
72	4.27	61.9	phosphatidylcholine
73	0.92	16.0	non n-3 FA
74	0.86	24.5	cholesterol_22
75	2.02	29.6	UFA
76	3.62	68.9	phosphatidylcholine
77	3.82	68.6	betaine
78	5.35	130.7	OF UFA
79	2.83	28.0	PUFA
80	3.97	71.0	lactate
81	3.05	50.7	taurine
82	3.28	38.9	taurine
83	3.36	44.1	glycine
84	1.30	23.2	lactate
85	3.25	55.3	betaine
86	3.21	56.2	phosphatidylcholine
87	3.01	39.3	creatine
88	3.84	56.8	creatine
89	1.27	32.3	FA
90	2.13	29.1	glutamine
91	2.28	32.0	valine,proline
92	2.44	34.0	glutamine
93	2.09	31.9	proline
94	7.30	131.7	phenylalanine
95	7.11	132.9	tyrosine
96	1.76	27.2	leucine
97	3.90	58.6	tyrosine
98	8.04	147.7	IMP
99	1.32	32.0	FA

[a] The peak number is consistent with that in **Figure S7**.

Table S8. Microorganism name list highlighted in the network analysis.

Microorganism Number[a]	Microorganism Name
MB_1	*Bacteria.Firmicutes.Bacilli.Lactobacillales.Carnobacteriaceae.Carnobacterium*
MB_2	*Bacteria.TM7.TM7.3.I025..*
MB_3	*Bacteria.Verrucomicrobia.Other.Other.Other.Other*
MB_4	*Bacteria.Actinobacteria.Acidimicrobiia.Acidimicrobiales.koll13.mixed*
MB_5	*Bacteria.Bacteroidetes.Cytophagia.Cytophagales.Cyclobacteriaceae.*
MB_6	*Bacteria.Proteobacteria.Betaproteobacteria.Other.Other.Other*
MB_7	*Bacteria.Proteobacteria.Gammaproteobacteria.Vibrionales.Other.Other*
MB_8	*Bacteria.Actinobacteria.Actinobacteria.Actinomycetales.Williamsiaceae.Williamsia*
MB_9	*Bacteria.Firmicutes.Bacilli.Lactobacillales.Enterococcaceae.Vagococcus*
MB_10	*Bacteria.Proteobacteria.Gammaproteobacteria.Alteromonadales.Alteromonadaceae.BD2.13*
MB_11	*Bacteria.Cyanobacteria.Nostocophycideae.Nostocales.Nostocaceae.Other*
MB_12	*Bacteria.Cyanobacteria.Synechococcophycideae.Synechococcales.Synechococcaceae.Other*
MB_13	*Bacteria.Firmicutes.Bacilli.Bacillales.Staphylococcaceae.Other*
MB_14	*Bacteria.Proteobacteria.Betaproteobacteria.Burkholderiales.Oxalobacteraceae.Massilia*
MB_15	*Bacteria.Proteobacteria.Alphaproteobacteria.Rhizobiales.Bradyrhizobiaceae.Bosea*
MB_16	*Bacteria.Proteobacteria.Gammaproteobacteria.Xanthomonadales.Xanthomonadaceae.Other*
MB_17	*Bacteria.Firmicutes.Bacilli.Lactobacillales.Aerococcaceae.Other*
MB_18	*Bacteria.Actinobacteria.Actinobacteria.Actinomycetales.Microbacteriaceae.Rathayibacter*
MB_19	*Bacteria.Bacteroidetes.Bacteroidia.Bacteroidales.Prevotellaceae.Prevotella*
MB_20	*Bacteria.Bacteroidetes.Flavobacteriia.Flavobacteriales..*
MB_21	*Bacteria.Proteobacteria.Alphaproteobacteria.Rickettsiales.mitochondria.*
MB_22	*Bacteria.Chloroflexi.Anaerolineae.Caldilineales.Caldilineaceae.Other*
MB_23	*Bacteria.Actinobacteria.Actinobacteria.Actinomycetales.Nocardioidaceae.*
MB_24	*Bacteria.Proteobacteria.Gammaproteobacteria.HOC36..*
MB_25	*Bacteria.Proteobacteria.Alphaproteobacteria.Rhizobiales.Xanthobacteraceae.Other*
MB_26	*Bacteria.Actinobacteria.Thermoleophilia.Solirubrobacterales..*
MB_27	*Bacteria.Planctomycetes.Planctomycetia.Gemmatales.Gemmataceae.Gemmata*
MB_28	*Bacteria.Actinobacteria.Actinobacteria.Actinomycetales.Actinomycetaceae.Varibaculum*
MB_29	*Bacteria.Actinobacteria.Actinobacteria.Actinomycetales.Nocardioidaceae.Propionicimonas*
MB_30	*Bacteria.Firmicutes.Clostridia.Clostridiales.Veillonellaceae.Veillonella*
MB_31	*Bacteria.Firmicutes.Bacilli.Other.Other.Other*
MB_32	*Bacteria.Chloroflexi.Anaerolineae.Caldilineales.Caldilineaceae.Caldilinea*

MB_33	*Bacteria.Firmicutes.Clostridia.Clostridiales..Tissierellaceae..Finegoldia*
MB_34	*Bacteria.Bacteroidetes.Other.Other.Other.Other*
MB_35	*Bacteria.Actinobacteria.Thermoleophilia...*
MB_36	*Bacteria.Firmicutes.Clostridia.Clostridiales.Lachnospiraceae.Other*
MB_37	*Archaea.Crenarchaeota.Thaumarchaeota.Nitrososphaerales.Nitrososphaeraceae.Candidatus.Nitrososphaera*
MB_38	*Unclassified.Other.Other.Other.Other.Other*
MB_39	*Bacteria.Proteobacteria.Gammaproteobacteria.Legionellales.Coxiellaceae.Rickettsiella*
MB_40	*Bacteria.Proteobacteria.Alphaproteobacteria.Rickettsiales.mitochondria.Thalassiosira*
MB_41	*Bacteria.Fusobacteria.Fusobacteriia.Fusobacteriales.Leptotrichiaceae.Leptotrichia*
MB_42	*Bacteria.Firmicutes.Clostridia.Clostridiales.Lachnospiraceae.Dorea*
MB_43	*Bacteria.Bacteroidetes.Flavobacteriia.Flavobacteriales..Weeksellaceae..Chryseobacterium*
MB_44	*Bacteria.Proteobacteria.Alphaproteobacteria.Rhizobiales.Phyllobacteriaceae.*
MB_45	*Bacteria.Chloroflexi.Anaerolineae.DRC31..*
MB_46	*Bacteria.Proteobacteria.Gammaproteobacteria.Alteromonadales.Other.Other*
MB_47	*Bacteria.Firmicutes.Bacilli.Lactobacillales.Enterococcaceae.Enterococcus*
MB_48	*Bacteria.WS2.SHA.109...*
MB_49	*Bacteria.Bacteroidetes..Rhodothermi...Rhodothermales..Other.Other*
MB_50	*Bacteria.TM7....*
MB_51	*Bacteria.TM7.TM7.3.I025.Rs.045.*
MB_52	*Bacteria.Acidobacteria..Chloracidobacteria..RB41.Other.Other*
MB_53	*Bacteria.Proteobacteria.Gammaproteobacteria.Pseudomonadales.Pseudomonadaceae.Pseudomonas*
MB_54	*Bacteria..Thermi..Deinococci.Deinococcales.Deinococcaceae.Other*
MB_55	*Bacteria.Proteobacteria.Alphaproteobacteria.Rhizobiales.Hyphomicrobiaceae.Polymorphum*
MB_56	*Bacteria.Actinobacteria.Actinobacteria.Actinomycetales.Nocardioidaceae.Nocardioides*
MB_57	*Bacteria.Proteobacteria.Gammaproteobacteria.Legionellales.Legionellaceae.Other*
MB_58	*Bacteria.TM7.TM7.3.Other.Other.Other*
MB_59	*Bacteria.Actinobacteria.Thermoleophilia.Gaiellales..*
MB_60	*Bacteria.Cyanobacteria.Oscillatoriophycideae.Oscillatoriales.Phormidiaceae.Phormidium*
MB_61	*Bacteria.Firmicutes.Clostridia.Clostridiales.Peptostreptococcaceae.*
MB_62	*Bacteria.Acidobacteria.Acidobacteria.6.CCU21..*
MB_63	*Bacteria.Chloroflexi.Gitt.GS.136...*
MB_64	*Bacteria.Firmicutes.Bacilli.Bacillales.Bacillaceae.Other*
MB_65	*Archaea.Euryarchaeota.Methanomicrobia.Methanosarcinales.Methanosarcinaceae.Methanosarcina*
MB_66	*Bacteria.Actinobacteria....*
MB_67	*Bacteria.Proteobacteria.Alphaproteobacteria.Rhizobiales.Methylocystaceae.Methylosinus*
MB_68	*Bacteria.Actinobacteria.Thermoleophilia.Solirubrobacterales.Conexibacteraceae.*

MB_69	*Bacteria.Chlamydiae.Chlamydiia.Chlamydiales.Parachlamydiaceae.*
MB_70	*Bacteria.Proteobacteria.Deltaproteobacteria.Desulfuromonadales.Desulfuromonadaceae.*
MB_71	*Bacteria.Proteobacteria.Gammaproteobacteria.Pasteurellales.Pasteurellaceae.Haemophilus*
MB_72	*Bacteria.Bacteroidetes.Bacteroidia.Bacteroidales.Bacteroidaceae.Bacteroides*
MB_73	*Bacteria.Actinobacteria.Coriobacteriia.Coriobacteriales.Coriobacteriaceae.Collinsella*
MB_74	*Bacteria.Proteobacteria.Alphaproteobacteria.Rhodospirillales.Acetobacteraceae.Roseomonas*
MB_75	*Bacteria.Proteobacteria.Gammaproteobacteria.Alteromonadales.Shewanellaceae.Shewanella*
MB_76	*Bacteria.Proteobacteria.Gammaproteobacteria.Enterobacteriales.Enterobacteriaceae.Yersinia*
MB_77	*Bacteria.Proteobacteria.Alphaproteobacteria.Sphingomonadales.Sphingomonadaceae.Sphingobium*
MB_78	*Bacteria.Proteobacteria.Gammaproteobacteria.Oceanospirillales.Oceanospirillaceae.Oleibacter*
MB_79	*Bacteria.Actinobacteria.Actinobacteria.Actinomycetales.Nocardioidaceae.Pimelobacter*
MB_80	*Bacteria.Bacteroidetes.Cytophagia.Cytophagales.Cytophagaceae.Hymenobacter*
MB_81	*Bacteria.Proteobacteria.Gammaproteobacteria.Alteromonadales.Alteromonadaceae.Glaciecola*
MB_82	*Bacteria.Proteobacteria.Gammaproteobacteria.Vibrionales.Vibrionaceae.Aliivibrio*
MB_83	*Bacteria.Actinobacteria.Actinobacteria.Actinomycetales.Nocardioidaceae.Other*
MB_84	*Bacteria.Firmicutes.Bacilli.Bacillales.Paenibacillaceae.Paenibacillus*
MB_85	*Bacteria.Actinobacteria.Acidimicrobiia.Acidimicrobiales.EB1017.*
MB_86	*Bacteria.Firmicutes.Clostridia.Clostridiales.Peptostreptococcaceae.Clostridium*
MB_87	*Bacteria.Proteobacteria.Deltaproteobacteria.Desulfobacterales.Desulfobacteraceae.Other*
MB_88	*Bacteria.Proteobacteria.Alphaproteobacteria.Rickettsiales..*
MB_89	*Bacteria.Proteobacteria.Gammaproteobacteria.Xanthomonadales.Sinobacteraceae.*
MB_90	*Bacteria.Proteobacteria.Alphaproteobacteria.Rhizobiales.Rhizobiaceae.Agrobacterium*
MB_91	*Bacteria.Proteobacteria.Gammaproteobacteria.Legionellales.Other.Other*
MB_92	*Bacteria.Proteobacteria.Deltaproteobacteria.Desulfobacterales.Desulfobacteraceae.Desulfococcus*
MB_93	*Bacteria.Cyanobacteria.Oscillatoriophycideae.Oscillatoriales.Phormidiaceae.Other*
MB_94	*Bacteria.Cyanobacteria.Chloroplast.Cercozoa..*
MB_95	*Bacteria.Actinobacteria.Actinobacteria.Actinomycetales.Micrococcaceae.Rothia*
MB_96	*Bacteria.Proteobacteria.Betaproteobacteria.Methylophilales.Methylophilaceae.Methylotenera*
MB_97	*Bacteria.Bacteroidetes.Cytophagia.Cytophagales.Cytophagaceae.*
MB_98	*Bacteria.Actinobacteria.Actinobacteria.Other.Other.Other*
MB_99	*Bacteria.Cyanobacteria.Chloroplast.Chlorophyta.Chlamydomonadaceae.*
MB_100	*Bacteria.Firmicutes.Clostridia.Clostridiales.Lachnospiraceae.Blautia*
MB_101	*Bacteria.Verrucomicrobia.Verrucomicrobiae.Verrucomicrobiales.Verrucomicrobiaceae.Persicirhabdus*
MB_102	*Bacteria.Proteobacteria.Alphaproteobacteria.Sphingomonadales.Sphingomonadaceae.Kaistobacter*
MB_103	*Bacteria.Actinobacteria.Actinobacteria.Actinomycetales.Brevibacteriaceae.Brevibacterium*
MB_104	*Bacteria.Chlamydiae.Chlamydiia.Chlamydiales.Other.Other*

MB_105	*Bacteria.Firmicutes.Clostridia.Clostridiales.Veillonellaceae.Megamonas*
MB_106	*Bacteria.Actinobacteria.Acidimicrobiia.Acidimicrobiales.koll13.*
MB_107	*Bacteria.Proteobacteria.Alphaproteobacteria.Rhodobacterales.Rhodobacteraceae.Paracoccus*
MB_108	*Bacteria.Proteobacteria.Alphaproteobacteria.Caulobacterales.Caulobacteraceae.Caulobacter*
MB_109	*Bacteria.Chloroflexi.Anaerolineae.A31.S47.*
MB_110	*Bacteria.Actinobacteria.Actinobacteria.Actinomycetales.Geodermatophilaceae.Other*
MB_111	*Bacteria.Firmicutes.Bacilli.Lactobacillales.Aerococcaceae.*
MB_112	*Bacteria.Actinobacteria.Acidimicrobiia.Acidimicrobiales.koll13.Other*
MB_113	*Bacteria.Proteobacteria.Other.Other.Other.Other*
MB_114	*Bacteria.Proteobacteria.Gammaproteobacteria.Alteromonadales.Alteromonadaceae.Other*
MB_115	*Bacteria.Firmicutes.Clostridia.Clostridiales.Clostridiaceae.Clostridium*
MB_116	*Bacteria.Verrucomicrobia.Verrucomicrobiae.Verrucomicrobiales.Verrucomicrobiaceae.Other*
MB_117	*Bacteria.Firmicutes.Clostridia.Clostridiales..Tissierellaceae..Peptoniphilus*
MB_118	*Bacteria.Firmicutes.Bacilli.Bacillales.Planococcaceae.Other*
MB_119	*Bacteria.Proteobacteria.Gammaproteobacteria.Alteromonadales.OM60.*
MB_120	*Bacteria.Proteobacteria.Gammaproteobacteria.Alteromonadales.Colwelliaceae.Thalassomonas*
MB_121	*Bacteria.Proteobacteria.Gammaproteobacteria.Alteromonadales..*
MB_122	*Bacteria.Firmicutes.Bacilli.Bacillales.Other.Other*
MB_123	*Bacteria.Proteobacteria.Alphaproteobacteria.Sphingomonadales.Other.Other*
MB_124	*Bacteria.Firmicutes.Bacilli.Turicibacterales.Turicibacteraceae.Turicibacter*
MB_125	*Bacteria.Verrucomicrobia.Verrucomicrobiae.Verrucomicrobiales.Verrucomicrobiaceae.Verrucomicrobium*
MB_126	*Bacteria.Actinobacteria.Actinobacteria.Actinomycetales.Cellulomonadaceae.Demequina*
MB_127	*Bacteria.Firmicutes.Clostridia.Clostridiales..Tissierellaceae..WAL_1855D*
MB_128	*Bacteria.Proteobacteria.Alphaproteobacteria.Other.Other.Other*
MB_129	*Bacteria.Proteobacteria.Gammaproteobacteria.Alteromonadales.Alteromonadaceae.Marinobacter*
MB_130	*Bacteria.Actinobacteria.Thermoleophilia.Solirubrobacterales.Other.Other*
MB_131	*Archaea.Crenarchaeota.MCG.pGrfC26..*
MB_132	*Bacteria.Verrucomicrobia.Verrucomicrobiae.Verrucomicrobiales.Verrucomicrobiaceae.Luteolibacter*
MB_133	*Bacteria.Cyanobacteria.Oscillatoriophycideae.Chroococcales.Other.Other*
MB_134	*Bacteria.Planctomycetes.Planctomycetia.Pirellulales.Pirellulaceae.Other*
MB_135	*Bacteria.Verrucomicrobia.Verrucomicrobiae.Verrucomicrobiales.Verrucomicrobiaceae.Rubritalea*
MB_136	*Bacteria.Proteobacteria.Alphaproteobacteria.Caulobacterales.Caulobacteraceae.*
MB_137	*Bacteria.Proteobacteria.Alphaproteobacteria.Rhodobacterales.Rhodobacteraceae.Phaeobacter*
MB_138	*Bacteria.Proteobacteria.Betaproteobacteria.Burkholderiales.Comamonadaceae.Acidovorax*
MB_139	*Bacteria.Cyanobacteria.Synechococcophycideae.Pseudanabaenales.Pseudanabaenaceae.Leptolyngbya*
MB_140	*Bacteria.Proteobacteria.Gammaproteobacteria.Legionellales.Francisellaceae.*

MB_141	*Bacteria.Proteobacteria.Gammaproteobacteria..Marinicellales...Marinicellaceae..*
MB_142	*Bacteria.WS5....*
MB_143	*Bacteria.Chloroflexi.Ellin6529...*
MB_144	*Bacteria.Proteobacteria.Deltaproteobacteria.Desulfobacterales.Desulfobulbaceae.*
MB_145	*Bacteria.Actinobacteria.Actinobacteria.Actinomycetales.Dermacoccaceae.Dermacoccus*
MB_146	*Bacteria.Proteobacteria.Alphaproteobacteria.Rhizobiales.Bradyrhizobiaceae.Balneimonas*
MB_147	*Bacteria.Actinobacteria.Actinobacteria.Actinomycetales.Microbacteriaceae.Leucobacter*
MB_148	*Bacteria.Actinobacteria.Actinobacteria.Actinomycetales.Other.Other*
MB_149	*Bacteria.Cyanobacteria.Chloroplast.Chlorophyta..*
MB_150	*Bacteria.Planctomycetes.Planctomycetia.Pirellulales.Pirellulaceae.Planctomycete*
MB_151	*Bacteria.Actinobacteria.Actinobacteria.Actinomycetales.Sporichthyaceae.*
MB_152	*Bacteria.Proteobacteria.Deltaproteobacteria.Spirobacillales..*
MB_153	*Bacteria.Bacteroidetes.Bacteroidia.Bacteroidales..Odoribacteraceae..Odoribacter*
MB_154	*Bacteria.Proteobacteria.Gammaproteobacteria.Methylococcales.Methylococcaceae.Methylocaldum*
MB_155	*Bacteria.Proteobacteria.Gammaproteobacteria.Enterobacteriales.Enterobacteriaceae.Escherichia*
MB_156	*Bacteria.Firmicutes.Clostridia.Clostridiales.Lachnospiraceae..Ruminococcus.*
MB_157	*Bacteria.Proteobacteria.Gammaproteobacteria.Legionellales.Legionellaceae.*
MB_158	*Bacteria.Proteobacteria.Gammaproteobacteria.Pseudomonadales.Moraxellaceae.Enhydrobacter*
MB_159	*Bacteria.Chloroflexi.Anaerolineae.Caldilineales.Caldilineaceae.*
MB_160	*Bacteria.Proteobacteria.Gammaproteobacteria.Legionellales.Coxiellaceae.Other*
MB_161	*Bacteria.Actinobacteria.Actinobacteria.Actinomycetales.Micrococcaceae.Micrococcus*
MB_162	*Bacteria.Proteobacteria.Betaproteobacteria.Burkholderiales.Comamonadaceae.Aquabacterium*
MB_163	*Bacteria.Proteobacteria.Betaproteobacteria.Methylophilales.Methylophilaceae.Other*
MB_164	*Bacteria.Proteobacteria.Gammaproteobacteria.Xanthomonadales.Xanthomonadaceae.Stenotrophomonas*
MB_165	*Bacteria.Proteobacteria.Alphaproteobacteria.Sphingomonadales.Sphingomonadaceae.Blastomonas*
MB_166	*Bacteria.Actinobacteria.Acidimicrobiia.Acidimicrobiales.Microthrixaceae.*
MB_167	*Bacteria.Actinobacteria.Acidimicrobiia.Acidimicrobiales.Other.Other*
MB_168	*Bacteria.Proteobacteria.Alphaproteobacteria.Rhodobacterales.Rhodobacteraceae.Anaerospora*
MB_169	*Bacteria.Actinobacteria.Actinobacteria.Bifidobacteriales.Bifidobacteriaceae.Bifidobacterium*
MB_170	*Bacteria.Proteobacteria.Gammaproteobacteria.Chromatiales..*
MB_171	*Bacteria.Bacteroidetes..Saprospirae...Saprospirales..Chitinophagaceae.Other*
MB_172	*Bacteria.Chloroflexi.Anaerolineae.GCA004..*
MB_173	*Bacteria.Proteobacteria.Alphaproteobacteria.Sphingomonadales.Sphingomonadaceae.Other*
MB_174	*Bacteria.Proteobacteria.Alphaproteobacteria.Rhizobiales.Phyllobacteriaceae.Mesorhizobium*
MB_175	*Bacteria.Armatimonadetes.Armatimonadia.Armatimonadales.Armatimonadaceae.*
MB_176	*Bacteria.Actinobacteria.Actinobacteria.Actinomycetales.Intrasporangiaceae.Other*

MB_177	*Bacteria.Cyanobacteria.Nostocophycideae.Nostocales.Nostocaceae.*
MB_178	*Bacteria.Proteobacteria.Gammaproteobacteria.Cardiobacteriales.Cardiobacteriaceae.Cardiobacterium*
MB_179	*Bacteria.Actinobacteria.Rubrobacteria.Rubrobacterales.Rubrobacteraceae.Rubrobacter*
MB_180	*Bacteria.Proteobacteria.Alphaproteobacteria.Rhizobiales.Hyphomicrobiaceae.Hyphomicrobium*
MB_181	*Bacteria.Actinobacteria.OPB41...*
MB_182	*Bacteria.Actinobacteria.Actinobacteria.Actinomycetales.Dermabacteraceae.Brachybacterium*
MB_183	*Bacteria.Firmicutes.Clostridia.Clostridiales.Other.Other*
MB_184	*Bacteria.Proteobacteria.Gammaproteobacteria.Pseudomonadales.Pseudomonadaceae.Other*
MB_185	*Bacteria.Actinobacteria.Actinobacteria.Actinomycetales.Microbacteriaceae.Other*
MB_186	*Bacteria.Proteobacteria.Alphaproteobacteria.Rhizobiales..*
MB_187	*Bacteria.Actinobacteria.Actinobacteria.Actinomycetales.Micromonosporaceae.Virgisporangium*
MB_188	*Bacteria.Proteobacteria.Gammaproteobacteria.Alteromonadales..Chromatiaceae..*
MB_189	*Bacteria.Actinobacteria.Acidimicrobiia.Acidimicrobiales..*
MB_190	*Bacteria.Verrucomicrobia..Spartobacteria...Chthoniobacterales...Chthoniobacteraceae..DA101*
MB_191	*Bacteria.Proteobacteria.Epsilonproteobacteria.Campylobacterales.Helicobacteraceae.*
MB_192	*Bacteria..Thermi..Deinococci.Deinococcales.Deinococcaceae.Deinococcus*
MB_193	*Bacteria.Tenericutes.Mollicutes...*
MB_194	*Bacteria.Proteobacteria.Deltaproteobacteria.Desulfobacterales.Desulfobulbaceae.Other*
MB_195	*Bacteria.Actinobacteria.Acidimicrobiia.Acidimicrobiales.C111.*
MB_196	*Bacteria.Proteobacteria.Alphaproteobacteria.Rhizobiales.Hyphomicrobiaceae.Other*
MB_197	*Bacteria.Proteobacteria.Betaproteobacteria.Burkholderiales.Oxalobacteraceae.Other*
MB_198	*Bacteria.Proteobacteria.Gammaproteobacteria.Oceanospirillales.Endozoicimonaceae.*
MB_199	*Bacteria.Actinobacteria.Actinobacteria.Actinomycetales.Micrococcaceae.Kocuria*
MB_200	*Bacteria.Proteobacteria.Alphaproteobacteria.Rhodobacterales.Rhodobacteraceae.Rubellimicrobium*
MB_201	*Bacteria.Firmicutes.Bacilli.Lactobacillales.Other.Other*
MB_202	*Bacteria.Firmicutes.Bacilli.Lactobacillales.Streptococcaceae.Streptococcus*
MB_203	*Bacteria.Firmicutes.Clostridia.Clostridiales..Tissierellaceae..Anaerococcus*
MB_204	*Bacteria.Proteobacteria.Alphaproteobacteria.Rhizobiales.Hyphomicrobiaceae.Devosia*
MB_205	*Bacteria.TM6.SJA.4...*
MB_206	*Bacteria.Bacteroidetes.Flavobacteriia.Flavobacteriales.Flavobacteriaceae.Other*
MB_207	*Bacteria.Proteobacteria.Gammaproteobacteria.34P16..*
MB_208	*Bacteria.Firmicutes.Clostridia.Clostridiales.Ruminococcaceae.Other*
MB_209	*Bacteria.Acidobacteria.Acidobacteria.6.iii1.15.mb2424.*
MB_210	*Bacteria.Proteobacteria.Alphaproteobacteria.Rhizobiales.Other.Other*
MB_211	*Bacteria.Firmicutes.Clostridia.Clostridiales.Clostridiaceae.Other*
MB_212	*Bacteria.Actinobacteria.Actinobacteria.Actinomycetales.Intrasporangiaceae.Janibacter*

MB_213	*Bacteria.Proteobacteria.Gammaproteobacteria.Pseudomonadales.Moraxellaceae.Psychrobacter*
MB_214	*Bacteria.Actinobacteria.Actinobacteria.Actinomycetales.Propionibacteriaceae.Propionibacterium*
MB_215	*Bacteria.Proteobacteria.Gammaproteobacteria.Vibrionales.Vibrionaceae.Vibrio*
MB_216	*Bacteria.Bacteroidetes.Flavobacteriia.Flavobacteriales.Flavobacteriaceae.Flavobacterium*
MB_217	*Bacteria.Actinobacteria.Actinobacteria.Actinomycetales.Mycobacteriaceae.Mycobacterium*
MB_218	*Bacteria.Proteobacteria.Alphaproteobacteria.Rickettsiales.mitochondria.Other*
MB_219	*Bacteria.Cyanobacteria.Synechococcophycideae.Synechococcales.Synechococcaceae.Synechococcus*
MB_220	*Bacteria..Thermi..Deinococci.Deinococcales.Trueperaceae.Truepera*
MB_221	*Bacteria.Planctomycetes.Planctomycetia.Planctomycetales.Planctomycetaceae.Planctomyces*
MB_222	*Bacteria.Acidobacteria..Chloracidobacteria..RB41.Ellin6075.*
MB_223	*Bacteria.Proteobacteria.Gammaproteobacteria.Enterobacteriales.Enterobacteriaceae.Other*
MB_224	*Bacteria.Acidobacteria..Chloracidobacteria..RB41..*
MB_225	*Bacteria.Proteobacteria.Gammaproteobacteria.Vibrionales.Vibrionaceae.Other*
MB_226	*Bacteria.Actinobacteria.Actinobacteria.Actinomycetales..*
MB_227	*Bacteria.Proteobacteria.Gammaproteobacteria.Other.Other.Other*
MB_228	*Bacteria.Firmicutes.Bacilli.Lactobacillales.Aerococcaceae.Alkalibacterium*
MB_229	*Bacteria.Other.Other.Other.Other.Other*
MB_230	*Bacteria.Chloroflexi.Anaerolineae.SBR1031.A4b.*
MB_231	*Bacteria.Fusobacteria.Fusobacteriia.Fusobacteriales.Fusobacteriaceae.Cetobacterium*
MB_232	*Bacteria.Proteobacteria.Alphaproteobacteria.Rhizobiales.Xanthobacteraceae.*
MB_233	*Bacteria.Firmicutes.Bacilli.Bacillales.Staphylococcaceae.Jeotgalicoccus*
MB_234	*Bacteria.Bacteroidetes.Flavobacteriia.Flavobacteriales..Weeksellaceae..Cloacibacterium*
MB_235	*Bacteria.Firmicutes.Bacilli.Bacillales.Bacillaceae.Bacillus*
MB_236	*Bacteria.Firmicutes.Clostridia.Clostridiales.Peptostreptococcaceae.Other*
MB_237	*Bacteria.Proteobacteria.Alphaproteobacteria.Caulobacterales.Caulobacteraceae.Other*
MB_238	*Bacteria.Cyanobacteria.Chloroplast.Streptophyta..*
MB_239	*Bacteria.Proteobacteria.Betaproteobacteria.Neisseriales.Neisseriaceae.*
MB_240	*Bacteria.Proteobacteria.Alphaproteobacteria.Rhodobacterales.Rhodobacteraceae.Other*
MB_241	*Bacteria.Proteobacteria.Betaproteobacteria.Burkholderiales.Comamonadaceae.Other*
MB_242	*Bacteria.Proteobacteria.Alphaproteobacteria.Rhizobiales.Bradyrhizobiaceae.Bradyrhizobium*
MB_243	*Bacteria.Cyanobacteria.Chloroplast.CAB.I..*
MB_244	*Bacteria.Planctomycetes.Planctomycetia.Pirellulales.Pirellulaceae.*
MB_245	*Bacteria.Proteobacteria.Alphaproteobacteria.Rhodospirillales.Rhodospirillaceae.Skermanella*
MB_246	*Bacteria.Proteobacteria.Gammaproteobacteria.Vibrionales.Vibrionaceae.Photobacterium*
MB_247	*Bacteria.Cyanobacteria.Chloroplast.Stramenopiles..*
MB_248	*Bacteria.Bacteroidetes..Saprospirae...Saprospirales..Chitinophagaceae.Sediminibacterium*

MB_249	*Bacteria.Proteobacteria.Alphaproteobacteria.Rhizobiales.Methylobacteriaceae.Methylobacterium*
MB_250	*Bacteria.Actinobacteria.Actinobacteria.Actinomycetales.Corynebacteriaceae.Corynebacterium*
MB_251	*Bacteria.Actinobacteria.Actinobacteria.Actinomycetales.Micrococcaceae.Other*
MB_252	*Bacteria.Proteobacteria.Alphaproteobacteria.Rhizobiales.Phyllobacteriaceae.Phyllobacterium*
MB_253	*Bacteria.Firmicutes.Bacilli.Bacillales.Staphylococcaceae.Staphylococcus*
MB_254	*Bacteria.Proteobacteria.Gammaproteobacteria.Pseudomonadales.Moraxellaceae.Acinetobacter*
MB_255	*Bacteria.Proteobacteria.Alphaproteobacteria.Rhizobiales.Bradyrhizobiaceae.Other*
MB_256	*Bacteria.Proteobacteria.Alphaproteobacteria.Rhizobiales.Phyllobacteriaceae.Other*
MB_257	*Bacteria.Actinobacteria.Actinobacteria.Actinomycetales.Micrococcaceae.Renibacterium*
MB_258	*Bacteria.Proteobacteria.Alphaproteobacteria.Sphingomonadales.Sphingomonadaceae.Sphingomonas*

[a] The number is consistent with that in **Figures 5** and **S5**.

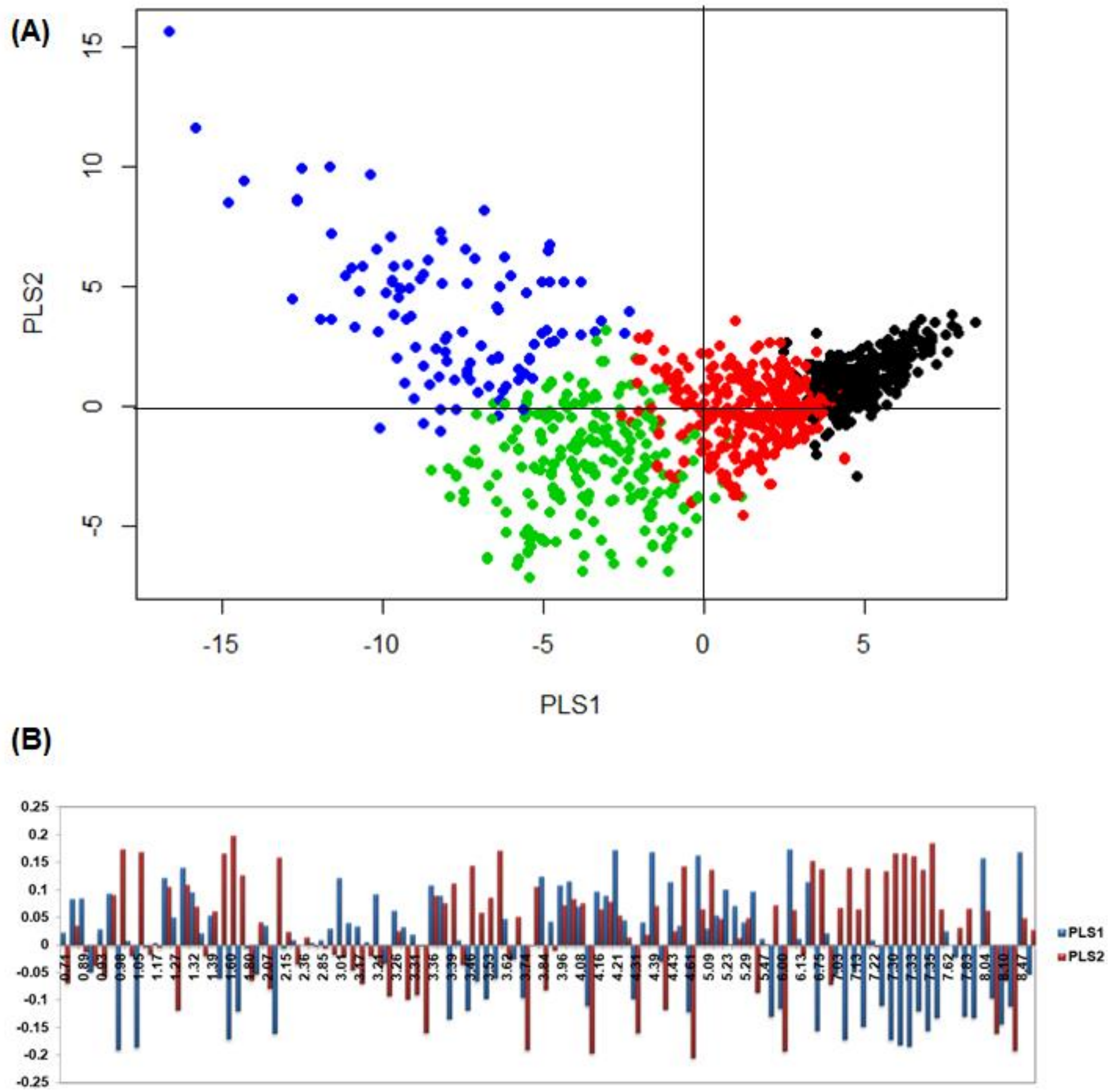

Figure S1. PLS-DA analysis of muscle NMR spectral data of yellowfin goby fish. **(A)** score plot;**(B)** loading plots of PLS1 and PLS2. The top 30 NMR peaks in the loading plots were assigned and summarized in Table S2. The color of groups was consistent with x-means clusters in Figures 2 and Tables S1.

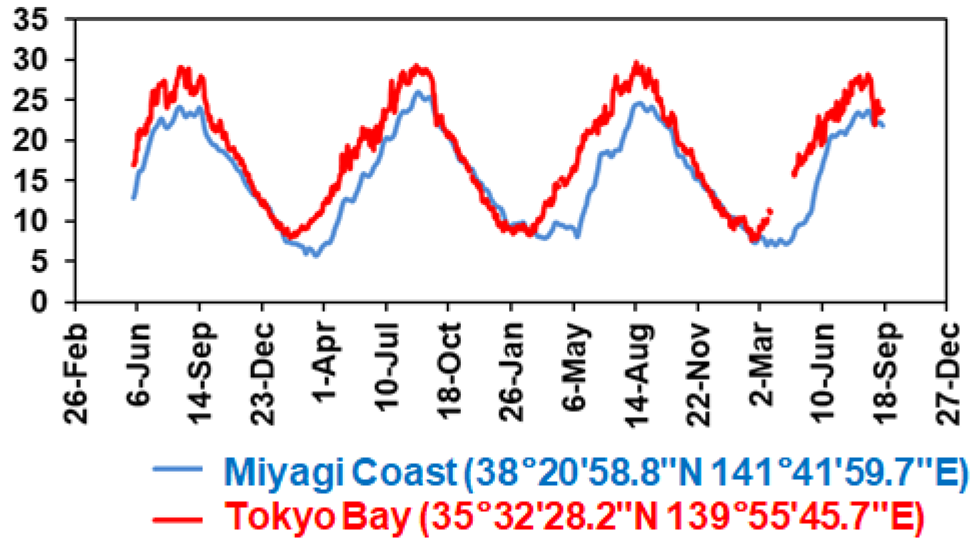

Figure S2. The available water temperature (upper layer) of Miyagi Coast and Tokyo Bay during the sampling period from 2011/05/31 to 2014/09/15. (Source: Ministry of Land, Infrastructure, Transport and Tourism. Kanto Regional Development Bureau. http://www.tbeic.go.jp/MonitoringPost/Top; Japan Meteorological Agency, http://www.jma-net.go.jp/sendai/wadai/umi/engan.html)

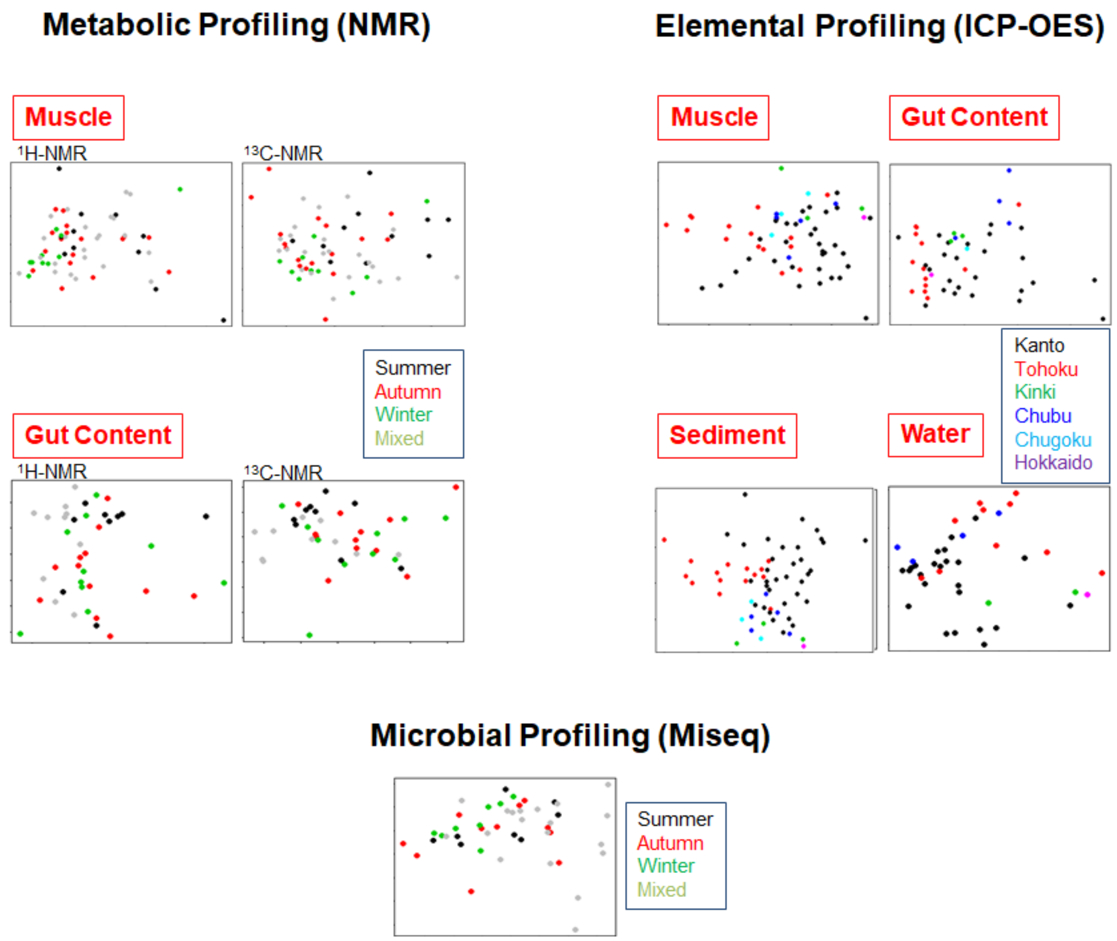

Figure S3. PCA score plots (PC1 vs. PC2) of NMR-based metabolic profiling, ICP-OES-based elemental profiling and Miseq-based microbial profiling of the living system of pooled yellowfin goby.

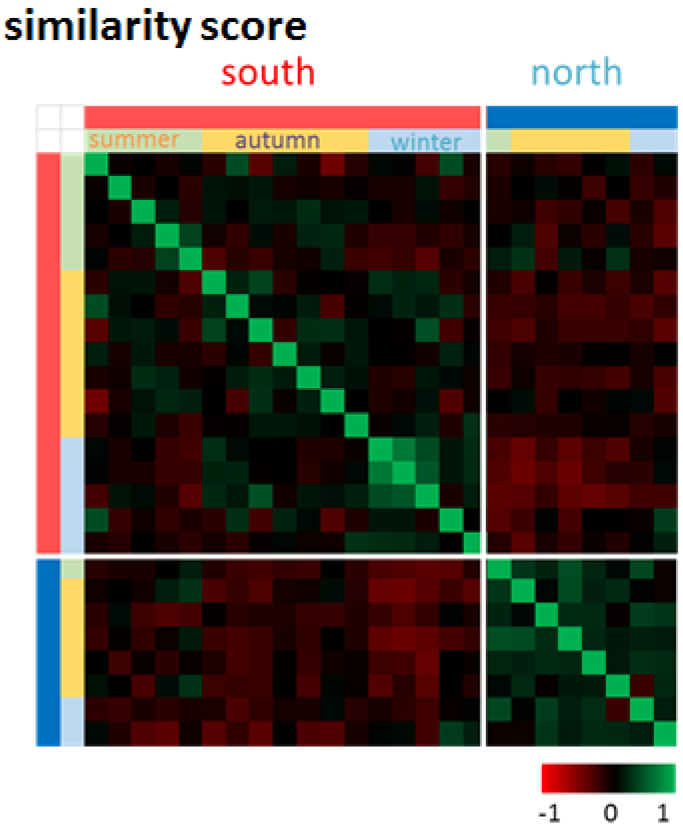

Figure S4. The similarity score of yellowfin goby ecosystems. The Spearman's correlations was calculated using the integrated datasets (yellowfin goby, symbiotic microbiota and inorganic environment).

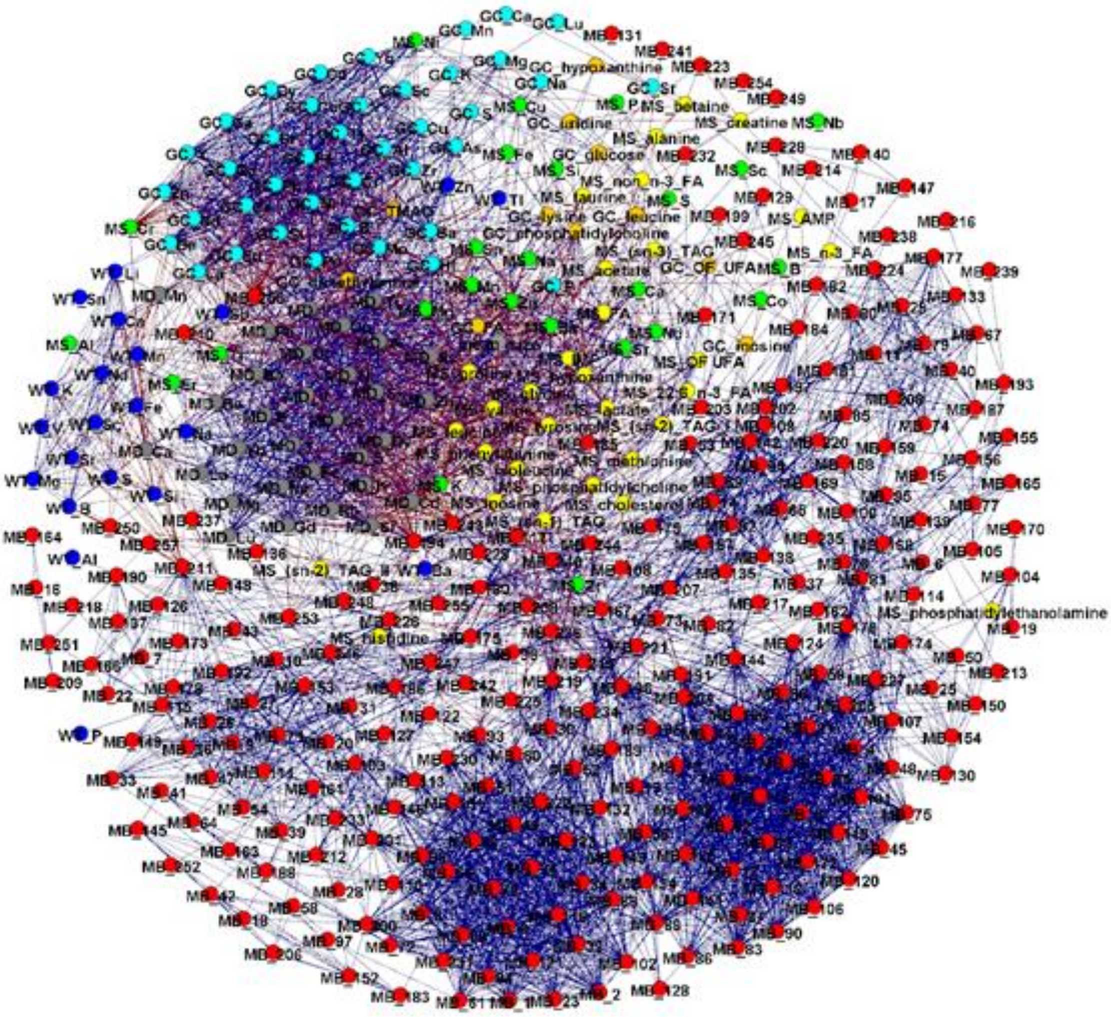

Figure S5. Correlation network analysis of metabolome, inonome and microbiome of the pooled yellowfin goby samples. Spearman's correlation coefficients (cor) between variants of integral data sets were calculated, and correlations with |cor| > 0.5 were selected as edges. MS, muscle; WT, water; MD, sediment; GT, gut content; MB, gut microbiota. The ID code of gut microorganism is consist with **Table S6**.

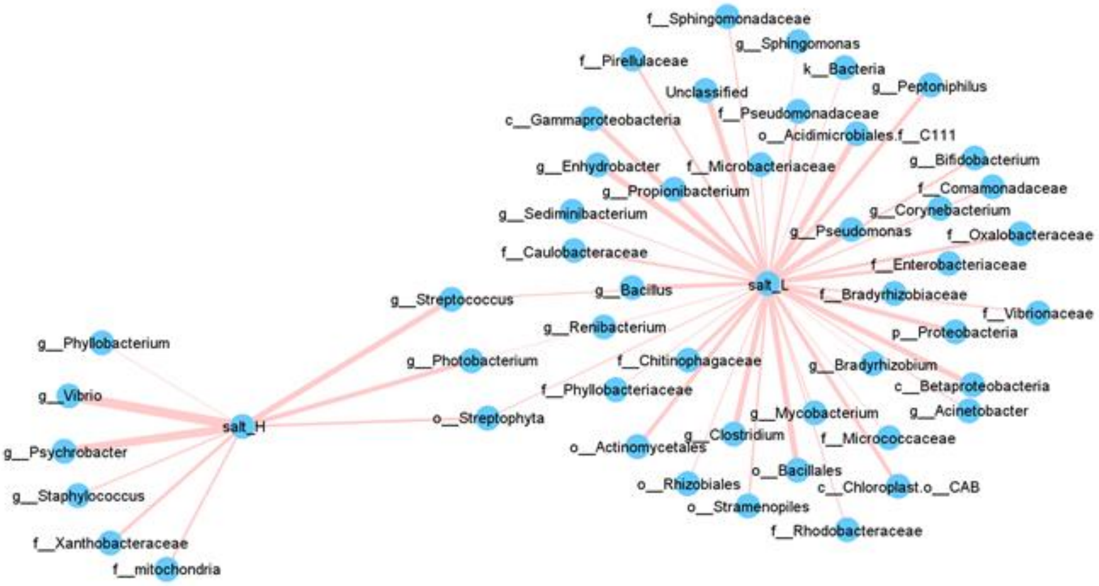

Figure S6. MBA network analyses of integral data sets obtained by pooled yellowfin goby, which partial highlighted by salinity with internal microbiota. Support = 0.625, confidence = 0.25, lift > 1. "_L" indicates metabolites at low level, and "_H" indicates metabolites at high level; "p_", "c_", "o_", "f_" and "g_" indicate Phylum, Class, Order, Family and Genus levels, respectively, which identified using NGS.

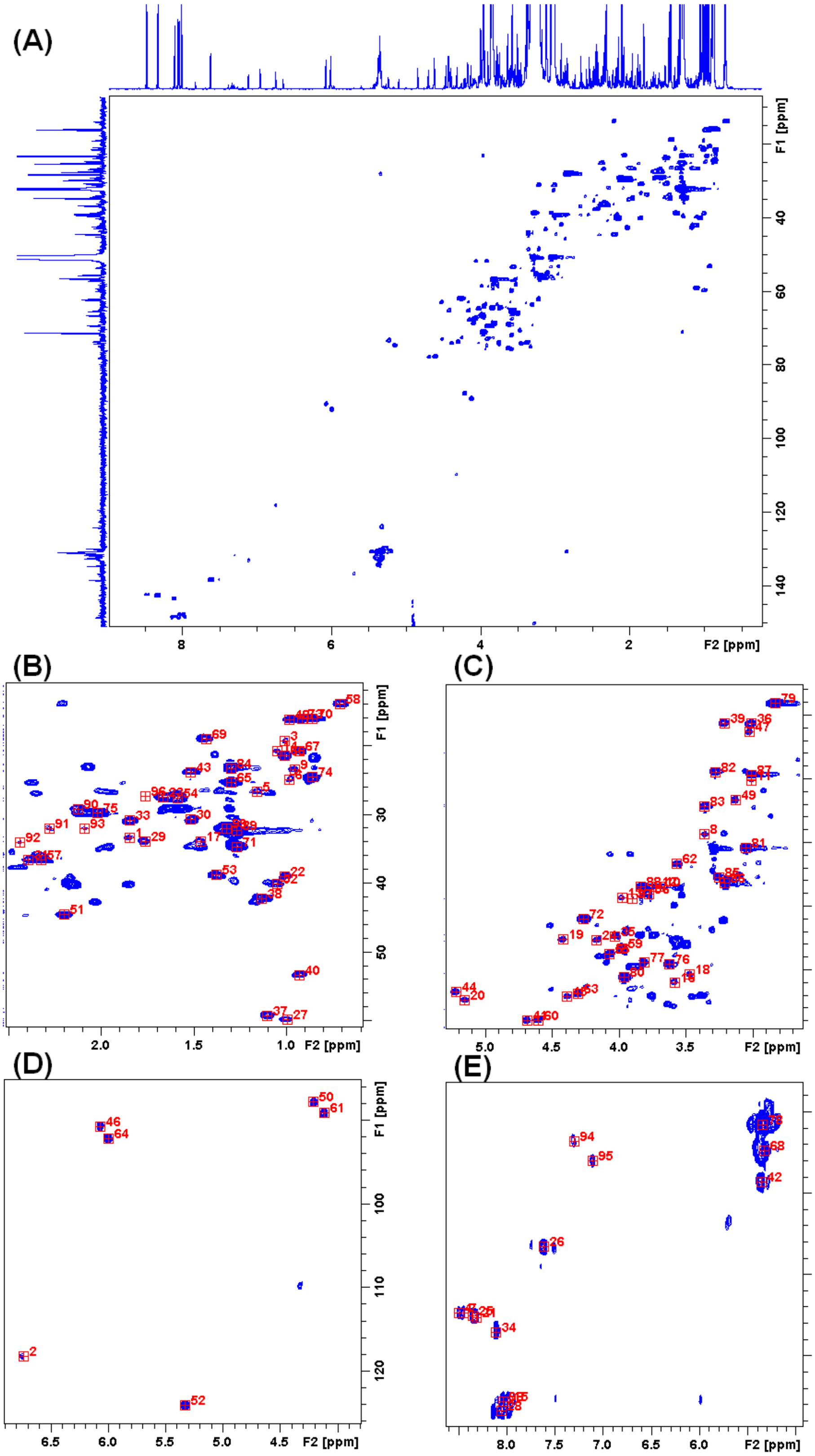

Figure S7. **(A)** ^{1}H-^{13}C HSQC spectrum and **(B)** ~ **(E)** expansions of yellowfin goby muscle extracts using methanol-d_4. The peak IDs are consistent with those in **Table S5**.

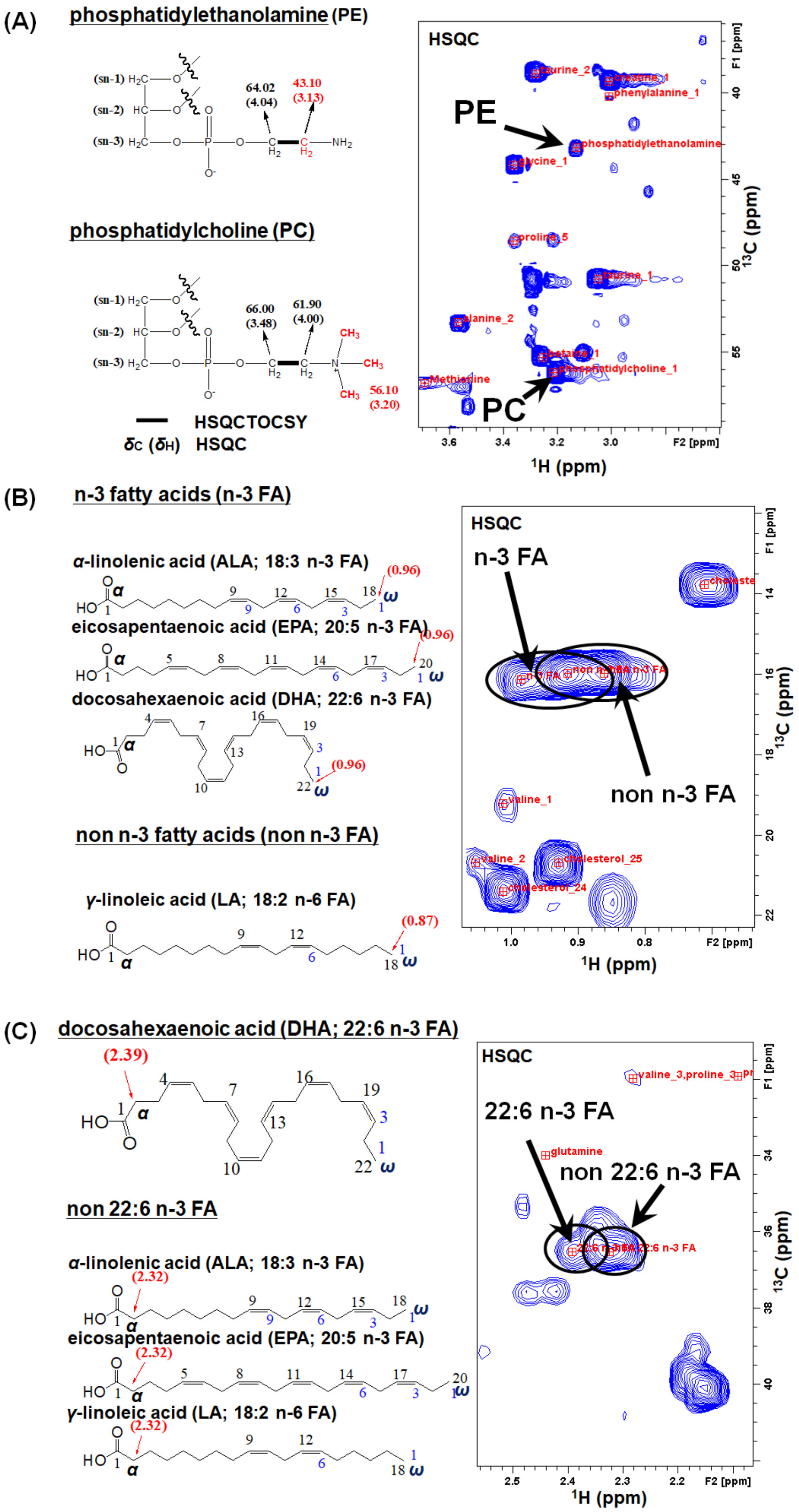

Figure S8. NMR signal annotation of lipids in yellowfin goby muscle extracts using methanol-d_4. Distinguishable signals between **(A)** phosphatidylethanolamine and

phosphatidylcholine; **(B)** n-3 fatty acids and non n-3 fatty acids; **(C)** 22:6 n-3 fatty acid and non 22:6 n-3 fatty acids.
